# The *FaAP2-11* Transcription Factor Putatively Regulates Cell Wall Remodeling and Stress-Acclimation Responses Under Drought and Salinity Stress

**DOI:** 10.3390/ijms27188203

**Published:** 2026-09-15

**Authors:** María Dolores Moreno-Recio, Facundo Spadoni-Revol, Sara Aguado-Delgado, Sara Posé, José A. Mercado, José Luis Caballero, Enriqueta Moyano, Francisco J. Molina-Hidalgo, Josefina P. Fernández-Moreno, Francisco J. Ruiz-Gómez, Juan Muñoz-Blanco, Rosario Blanco-Portales

**Affiliations:** 1 Departamento de Bioquímica y Biología Molecular, Edificio Severo Ochoa, Universidad de Córdoba, Campus Universitario de Rabanales, 14071 Córdoba, Spain; b42morem@uco.es (M.D.M.-R.); bb2spref@uco.es (F.S.-R.); b62agdes@uco.es (S.A.-D.); bb1carej@uco.es (J.L.C.); bb2mocae@uco.es (E.M.); b52mohif@uco.es (F.J.M.-H.); jpfernandez@uco.es (J.P.F.-M.); 2 Instituto de Hortofruticultura Subtropical y Mediterránea ‘La Mayora’ (IHSM-UMA-CSIC), Departamento de Botánica y Fisiología Vegetal, Universidad de Málaga, 29071 Málaga, Spain; sarapose@uma.es (S.P.); mercado@uma.es (J.A.M.); 3 Instituto Interuniversitario del Sistema Tierra de Andalucía (IISTA), Córdoba Headquarters, Campus de Rabanales, Edificio Leonardo Da Vinci, Crta. N-IV Km. 396, 14071 Córdoba, Spain; g72rugof@uco.es

**Keywords:** *Fragaria × ananassa*, AP2/ERF transcription factors, *FaAP2-11*, abiotic stress, drought, salinity, cell wall remodeling, water-use efficiency

## Abstract

APETALA2/ethylene-responsive factor (AP2/ERF) transcription factors are key regula-tors of plant responses to abiotic stress, integrating environmental and hormonal signals into coordinated transcriptional programs. RNA-seq analysis of strawberry (*Fragaria × ananassa*) leaves and roots subjected to drought and salinity identified 58 differentially expressed *FaAP2/ERF* genes. Promoter analysis revealed *cis*-regulatory elements associated with abscisic acid, jasmonate, ethylene, salicylic acid, and drought-responsive signaling. Among these genes, *FaAP2-11* was selected for functional characterization because of its strong response to both stresses. Expression profiling revealed preferential transcript accumulation in immature achenes and sepals, while *FaAP2-11* expression was induced by oxidative and nitrosative signals and stress-related phytohormones. Heterologous overexpression of *FaAP2-11* in *Nicotiana benthamiana* enhanced photosynthetic performance and intrinsic water-use efficiency under osmotic and salinity stress. Transcriptomic analysis revealed extensive reprogramming affecting hormone signaling, cell wall remodeling, primary metabolism, and redox-related processes. This response was characterized by repression of genes involved in cell wall modification and cuticular wax biosynthesis, together with selective upregulation of lignification-associated genes. Collectively, these findings suggest that *FaAP2-11* contributes to coordinating transcriptional and physiological responses associated with abiotic-stress acclimation and supports its potential as a candidate gene for improving drought and salinity resilience in strawberry.

## 1. Introduction

Drought and soil salinity are among the most widespread abiotic constraints limiting plant growth, development and crop productivity. Their impact is expected to intensify under climate-change scenarios, particularly in agricultural regions exposed to altered precipitation patterns, increased evapotranspiration and irrigation-dependent cultivation. Although drought and salinity differ in their primary causes, they share an early osmotic component resulting from reduced external water potential. In drought, this leads directly to cellular dehydration and hydraulic limitation, whereas salinity additionally imposes a later ionic phase caused by toxic Na^+^ and Cl^−^ accumulation, disruption of K^+^ homeostasis and nutrient imbalance. Both stresses converge on common physiological bottlenecks, including stomatal regulation, growth restriction, osmotic adjustment, reactive oxygen species (ROS) homeostasis, membrane protection and metabolic reprogramming. These responses require extensive transcriptional regulation to balance stress tolerance with continued development and yield maintenance [1,2,3].

Transcription factors are central components of this adaptive reprogramming because they connect stress perception, hormone signaling, signal-transduction pathways and chromatin-dependent regulation with the activation or repression of downstream stress-responsive genes. Beyond the AP2/ERF superfamily, several other transcription factors (TF) families, including NAC, WRKY, MYB, bZIP, and bHLH, play important roles in the transcriptional reprogramming underlying plant responses to abiotic stresses. These TFs participate in interconnected regulatory processes involving hormonal signaling, osmotic adjustment, ion homeostasis, reactive oxygen species (ROS) homeostasis, and the expression of stress-protective genes. In *Fragaria vesca*, overexpression of FvNAC29 in *Arabidopsis* thaliana enhanced tolerance to salt and cold stress and was associated with increased antioxidant capacity and altered expression of stress-responsive genes [4]. Similarly, the *Malus baccata* WRKY transcription factors MbWRKY3 and MbWRKY50 have been functionally associated with abiotic stress responses: MbWRKY3 enhanced drought tolerance when heterologously expressed in tobacco [5], whereas MbWRKY50 improved drought and cold tolerance in transgenic tomato plants, partly through enhanced antioxidant capacity and ROS-scavenging activity [6]. Moreover, heterologous expression of the grapevine MYB transcription factor VhMYB15 increased drought and salinity tolerance in *Arabidopsis*, accompanied by changes in stress-related physiological and molecular responses [7]. Together, these studies illustrate the contribution of diverse TF families to the complex transcriptional networks that coordinate plant acclimation to abiotic stress and provide a broader context for understanding the specific roles of AP2/ERF transcription factors in drought and salinity responses.

Among plant transcription factor families, APETALA2/ethylene-responsive factor (AP2/ERF) proteins constitute one of the largest and most functionally diverse superfamilies [8,9]. AP2/ERF transcription factors are defined by the conserved AP2 DNA-binding domain and include several major lineages, notably the AP2, ERF, DREB, RAV and Soloist groups [10]. Their regulatory versatility is partly explained by their ability to recognize stress-related *cis*-regulatory elements. DREB-type proteins classically bind dehydration-responsive DRE/CRT/C-repeat elements (A/GCCGAC), whereas many ERF proteins recognize GCC-box elements (AGCCGCC), which are associated with ethylene-responsive transcription [11]. However, these functional categories are not absolute, since promoter context, flanking sequences, chromatin state, protein partners and hormone crosstalk can broaden or modify DNA-binding specificity and transcriptional outcomes [9].

The ERF and DREB branches are particularly relevant to abiotic stress responses and have expanded substantially during plant evolution, generating species-specific repertoires that contribute to distinct adaptive strategies [12,13]. AP2/ERF proteins act as major regulatory hubs within drought and salinity gene regulatory networks. They translate environmental and hormonal cues into coordinated transcriptional outputs involving osmoprotective proteins, late embryogenesis abundant proteins, dehydrins, compatible-solute biosynthesis enzymes, antioxidant systems, membrane-stabilizing factors, cell wall remodeling components and ion-transport machinery [9,10].

In drought responses, AP2/ERF transcription factors participate in both ABA (abscisic acid)-dependent and ABA-independent regulatory pathways. Drought rapidly promotes ABA accumulation, leading to stomatal closure, osmotic adjustment, growth restriction and activation of protective gene expression. In parallel, ABA-independent DREB/CRT modules, together with extensive crosstalk involving jasmonate, ethylene, calcium, ROS and protein kinase signalling, contribute to the complexity of AP2/ERF-mediated stress regulation [9,10]. Canonical DREB/CBF regulators illustrate this regulatory framework. For example, overexpression of *Arabidopsis* DREB1A/CBF3 activates a broad suite of stress-responsive genes and enhances tolerance to drought and salinity, although constitutive expression frequently results in growth retardation [13]. These observations established two important concepts: AP2/ERF transcription factors can function as master regulators of abiotic-stress responses, but their activity must be tightly controlled in terms of timing, tissue specificity and expression level to minimize detrimental pleiotropic effects [13,14,15].

DREB2A provides another well-characterized example of AP2/ERF-mediated drought regulation. It activates dehydration-responsive genes through DRE/CRT-dependent mechanisms [16], but its activity is regulated at multiple levels, including transcriptional control, protein regulatory domains and post-translational mechanisms affecting protein activation and stability [17,18]. The presence of ABA-responsive elements within the DREB2A promoter, together with extensive crosstalk between ABA-dependent and ABA-independent signalling pathways, further links DREB2A to ABA-centred stress responses [19,20]. In addition, ubiquitin-mediated degradation by the RING E3 ligases DRIP1 and DRIP2 negatively regulates DREB2A stability and activity, providing an additional layer of control over drought-responsive gene expression [21]. More broadly, this example illustrates that AP2/ERF transcription factors are subject to complex multilayered regulation involving transcriptional regulation, phosphorylation, SUMOylation, ubiquitination, protein–protein interactions and integration of hormonal and redox signalling pathways [12,14].

AP2/ERF-dependent drought tolerance extends beyond the activation of classical stress-protective genes. Increasing evidence indicates that these transcription factors also reprogram developmental traits influencing water acquisition, transport and conservation. They participate in the regulation of root-system architecture, xylem development, cell wall remodeling and hydraulic performance, thereby contributing to plant adaptation under water deficit [22,23,24]. Representative examples include rice OsERF71, which promotes root architectural changes associated with drought resistance [22]; maize ZmERF21, which enhances seedling drought tolerance through the regulation of hormone- and stress-responsive genes [23]; and poplar PtoERF15, which improves drought tolerance via jasmonic-acid-mediated signaling [24]. Collectively, these studies reinforce the view that AP2/ERF transcription factors frequently operate as integrators of developmental and hormonal networks rather than as simple on/off regulators of stress-responsive genes [8,20].

Salinity tolerance requires the integration of osmotic and oxidative stress responses together with the maintenance of ion homeostasis. Plants must limit Na^+^ entry and long-distance transport, promote vacuolar sequestration, maintain K^+^ acquisition and preserve cellular osmotic balance [25,26]. AP2/ERF transcription factors contribute to these adaptive processes by coordinating hormone signaling, antioxidant capacity, membrane protection and the regulation of ion-transport-associated genes [12,13]. Representative examples include rice OsERF19, which enhances salt tolerance and ABA sensitivity through the regulation of genes involved in ABA metabolism/signaling and ion homeostasis, including OsNHX1 and OsHKT6 [26], whereas OsERF106MZ negatively affects shoot growth and salinity tolerance [27]. Together, these studies demonstrate that AP2/ERF transcription factors can function either as positive or negative regulators of salinity tolerance depending on gene identity, regulatory context and downstream transcriptional networks [28].

Similar functional diversity has been reported in woody and horticultural species, further illustrating the versatility of AP2/ERF transcription factors during salinity adaptation. In apple, MdERF023 acts as a negative regulator of salt tolerance by suppressing ABA signaling and downregulating ion-homeostasis genes such as MdSOS2 and MdAKT1, thereby impairing Na^+^ extrusion and K^+^ uptake [29]. In contrast, Populus PagERF021 enhances salt tolerance by promoting ROS scavenging and reducing membrane damage [30]. Likewise, TINY-like AP2/ERF proteins integrate drought-responsive gene activation with ABA-mediated stomatal regulation and brassinosteroid-controlled growth [14]. In strawberry, overexpression of FaTINY2 in *Arabidopsis* reduced ROS and malondialdehyde accumulation, increased proline and chlorophyll contents, enhanced CAT, SOD and POD activities, and improved tolerance to both salinity and cold stress [31]. Collectively, these studies highlight the remarkable functional versatility of AP2/ERF transcription factors across plant species.

Together, these findings support a model in which AP2/ERF transcription factors operate at multiple regulatory levels during salinity adaptation, including the modulation of hormone biosynthesis and signaling, regulation of ion transport and homeostasis, reinforcement of antioxidant systems and protection of cellular membranes [8,9,25].

Overall, the available evidence identifies AP2/ERF transcription factors as central integrators of drought and salinity signaling. They coordinate osmotic and ionic stress perception with hormonal regulation, post-translational control and downstream protective gene expression. Their ability to integrate stress-acclimation mechanisms with developmental programmes makes them attractive targets for crop improvement [32]. However, successful application in breeding and biotechnology requires a detailed understanding of their tissue specificity, inducibility, regulatory networks and potential pleiotropic effects. In strawberry and other horticultural crops, characterizing AP2/ERF expression patterns under drought, salinity and hormone treatments may therefore provide a mechanistic framework for identifying key regulators of cross-stress resilience and developing better adapted cultivars to water limitation and saline environments [33,34].

Within this context, transcriptomic analysis provides a powerful approach for identifying AP2/ERF genes associated with osmotic-stress adaptation. Comparative transcriptomic analyses between control plants and plants subjected to PEG (polyethylene glycol)-induced drought-like stress enable the identification of both tissue-specific and shared transcriptional responses in leaves and roots. In *Fragaria × ananassa* cv. Chandler, this strategy offers an opportunity to identify AP2/ERF transcription factors potentially involved in osmoprotection, ROS detoxification, hormone crosstalk, membrane stability, developmental adjustment and ion-homeostasis-related processes, thereby providing a framework for the functional characterization of candidate regulators such as *FaAP2-11* [29,30,31].

## 2. Results and Discussion

To gain insight into the role of APETALA2/ethylene-responsive factor (AP2/ERF) transcription factors in strawberry responses to drought and salinity stress, RNA-seq datasets generated from independent analyses of leaf and root tissues of plants subjected to drought-like osmotic stress (PEG treatment) and salt stress (NaCl treatment) were examined. A total of 58 FaAP2/ERF genes displaying differential expression under one or both stress conditions were identified and selected for subsequent phylogenetic, expression and functional analyses (Supplementary Table S1).

### 2.1. Phylogenetic Classification of AP2/ERF Transcription Factors Identified by RNA-Seq

**Figure 1:**
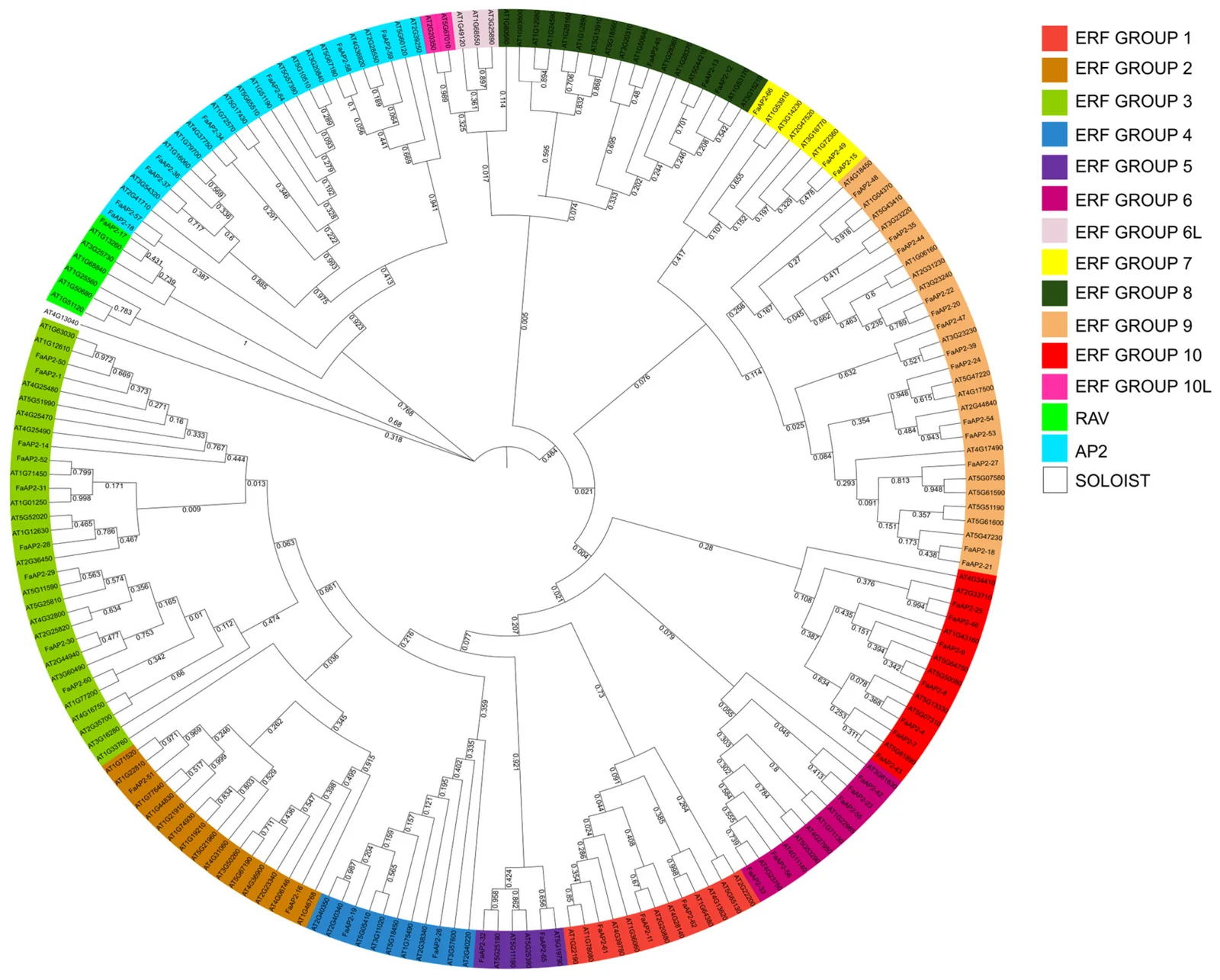
Phylogenetic analysis of AP2/ERF transcription factors identified from RNA-seq datasets under drought and salt stress conditions. The tree was constructed using AP2/ERF protein sequences from *Arabidopsis thaliana* and the identified FaAP2 proteins. Based on their phylogenetic relationships, *FaAP2* genes were classified into the AP2, RAV, and ERF subfamilies. The ERF members were further divided into ten groups (ERF I–X) according to the classification proposed by [6]. Bootstrap values are shown at the corresponding nodes. Different colors indicate the major AP2/ERF groups.

Members of the AP2/ERF superfamily are classified into four major subfamilies—AP2, ERF, RAV, and Soloist—according to the composition of their conserved AP2 DNA-binding domains and their evolutionary relationships. To classify the transcription factors identified through RNA-seq analysis, a phylogenetic tree was constructed using *Arabidopsis thaliana* AP2/ERF proteins as reference sequences. Based on this analysis, the identified genes were assigned to the corresponding AP2, ERF, RAV, and Soloist subfamilies previously described for the AP2/ERF family [10] (Figure 1).

All 58 proteins contained the characteristic AP2 DNA-binding domain and are therefore referred to hereafter using the unified FaAP2 nomenclature, irrespective of their assignment to the AP2, ERF, RAV or Soloist subfamilies (Supplementary Figure S1). Based on the phylogenetic analysis, eight genes were classified within the AP2 subfamily, namely *FaAP2-34*, *FaAP2-36*, *FaAP2-37*, *FaAP2-38*, *FaAP2-57*, *FaAP2-58*, *FaAP2-59*, and *FaAP2-64*. These genes are clustered with characteristic members of the AP2 subfamily from *A. thaliana*, forming an independent clade within the AP2/ERF superfamily. In addition, *FaAP2-17* was identified as the sole representative of the RAV subfamily among the analyzed genes.

The remaining 49 genes belonged to the ERF subfamily and were distributed across the ten canonical ERF groups (ERF I–X). Specifically, ERF I contained *FaAP2-11*, *FaAP2-61*, and *FaAP2-62*, ERF II comprised *FaAP2-16* and *FaAP2-51*, whereas ERF III included *FaAP2-1*, *FaAP2-14*, *FaAP2-28*, *FaAP2-29*, *FaAP2-30*, *FaAP2-31*, *FaAP2-50*, *FaAP2-52*, and *FaAP2-60*. *FaAP2-19* and *FaAP2-26* were assigned to ERF IV, while *FaAP2-32* and *FaAP2-65* belonged to ERF V. ERF VI included *FaAP2-23*, *FaAP2-33*, *FaAP2-42*, *FaAP2-55*, and *FaAP2-56.* ERF VII comprised *FaAP2-66*, *FaAP2-15*, and *FaAP2-49*, whereas *FaAP2-12*, *FaAP2-13*, and *FaAP2-45* clustered within ERF VIII. ERF IX was the largest group, containing *FaAP2-18*, *FaAP2-20*, *FaAP2-21*, *FaAP2-22*, *FaAP2-24*, *FaAP2-27*, *FaAP2-35*, *FaAP2-39*, *FaAP2-44*, *FaAP2-47*, *FaAP2-48*, *FaAP2-53*, and *FaAP2-54*. Finally, ERF X consisted of *FaAP2-4*, *FaAP2-7*, *FaAP2-8*, *FaAP2-9*, *FaAP2-25*, *FaAP2-43* and *FaAP2-46*.

Among the identified genes, the ERF subfamily predominated, consistent with the organization previously reported for the AP2/ERF superfamily in *A. thaliana* [10]. Among the identified genes, the ERF subfamily was predominant, accounting for 49 of the 58 analyzed genes and encompassing all ten established phylogenetic groups. ERF III and ERF IX were the most highly represented groups, containing nine and thirteen members, respectively, whereas the AP2 and RAV subfamilies were represented by eight and one gene, respectively. The predominance of ERF genes is particularly relevant because the analyzed sequences were identified exclusively from RNA-seq datasets generated under drought and salt stress conditions. Members of the ERF family have been widely reported as key regulators of abiotic stress responses, participating in both ABA-dependent and ABA-independent signaling pathways and modulating the expression of genes associated with drought, salinity, and osmotic stress tolerance [9,13,14]. Therefore, the abundance of *ERF* genes observed in this study suggests that these transcription factors may play a central role in the molecular mechanisms underlying drought and salt stress responses.

### 2.2. Predicted Subcellular Localization of FaAP2/ERF Proteins

Subcellular localization predictions indicated that most FaAP2 proteins are likely targeted to the nucleus, consistent with their expected role as transcription factors [9,14] (Supplementary Table S2). Nuclear localization signals were identified in 43 of the 58 proteins analyzed (74.1%), supporting the predominantly nuclear localization expected for this transcription factor family.

Only three proteins were predicted to contain organellar targeting signals. *FaAP2-15* and *FaAP2-45* were predicted to contain chloroplast transit peptides together with nuclear localization signals, suggesting potential dual targeting to the nucleus and chloroplast. Similar dual-targeting mechanisms have been reported for a limited number of plant regulatory proteins involved in plastid-to-nucleus communication [35]. In contrast, *FaAP2-32* displayed a strong mitochondrial targeting signal but no detectable nuclear localization signal, suggesting a potentially atypical localization pattern that requires experimental validation before any functional interpretation.

These predictions should nevertheless be interpreted with caution, since organellar targeting signals may reflect annotation inaccuracies, incomplete protein models or limitations of prediction algorithms. Therefore, experimental validation using fluorescent protein fusions or immunolocalization approaches will be required to confirm the actual subcellular localization of these proteins. Nevertheless, the predominance of predicted nuclear localization is consistent with the expected function of these proteins as transcriptional regulators and provides additional support for their annotation as members of the AP2/ERF superfamily.

### 2.3. Synteny Patterns of AP2/ERF Loci Between Fragaria vesca and Fragaria × ananassa

**Figure 2:**
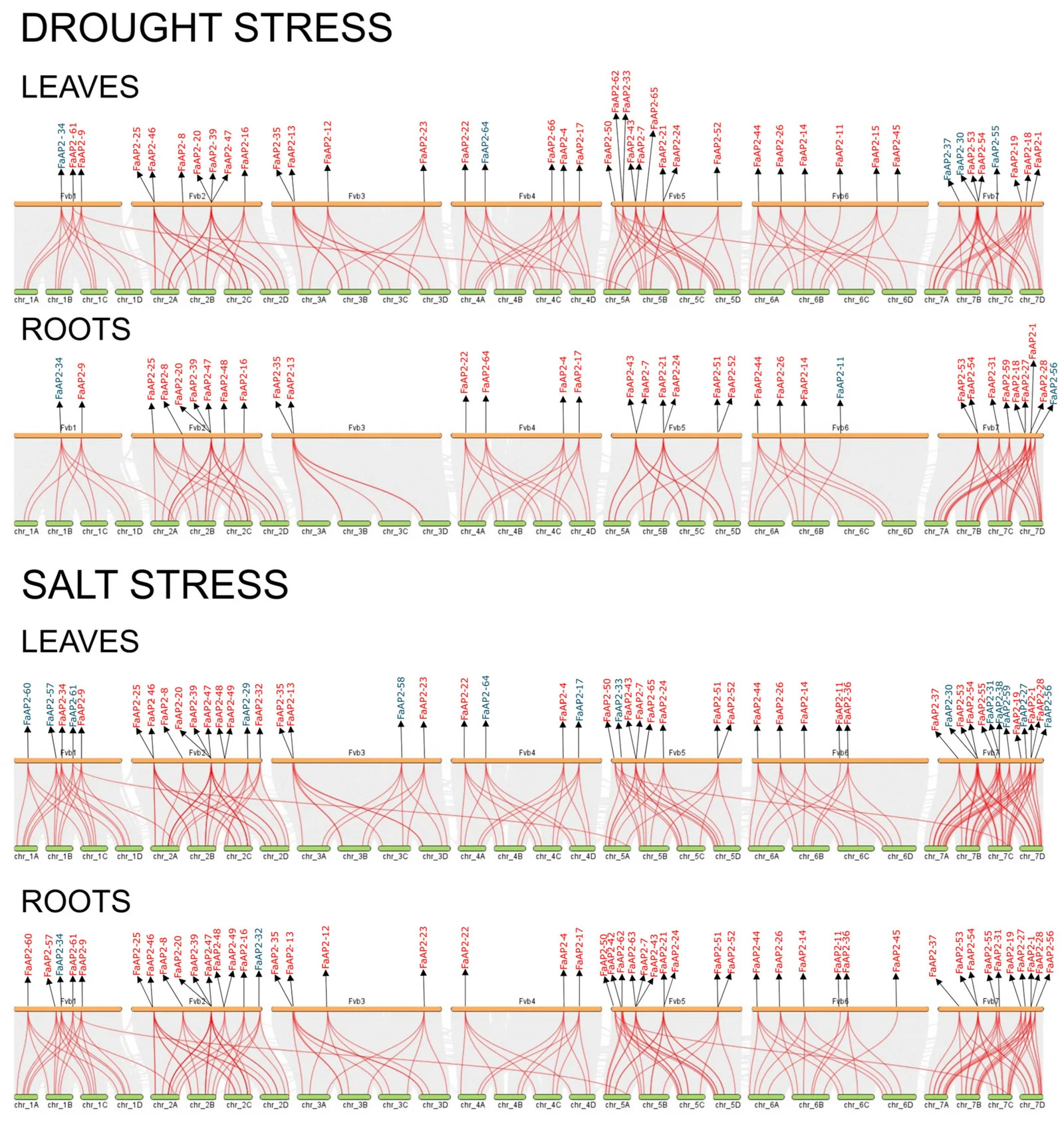
Synteny analysis of *AP2*-type genes between *Fragaria vesca* and *Fragaria × ananassa* under drought and salt stress conditions in leaves and roots. Lines connect orthologous *AP2* genes identified through collinearity analysis. Differentially expressed genes are shown in red (upregulated) and blue (downregulated).

Synteny analysis between the diploid genome of *Fragaria vesca* and the octoploid genome of *Fragaria × ananassa* showed that most AP2/ERF loci retained the expected four-homeolog (A–D) correspondence within the matching homeologous chromosome groups (Figure 2). Nevertheless, a substantial proportion of loci deviated from this canonical pattern through partial homeolog retention, apparent fractionation and/or non-canonical chromosomal placement, indicating that *AP2/ERF loci* have experienced heterogeneous evolutionary trajectories during octoploid genome evolution.

Several *loci* were represented by only two or three homeologous copies rather than the expected four. Such patterns may reflect genuine homeolog loss following polyploidization, incomplete genome annotation, failure to detect highly diverged copies, or methodological constraints associated with synteny detection, such as collinearity-block filtering. In addition, regions containing several neighbouring *AP2/ERF* genes in *F. vesca* frequently displayed dense syntenic connections in the octoploid genome, suggesting the presence of conserved gene clusters that probably originated through tandem or local duplication events prior to or following polyploidization.

Representative examples illustrate the diversity of these structural configurations. Some *loci* retained the complete four-homeolog complement while showing evidence of additional rearrangements. For example, *FaAP2-34* exhibited the expected A-, B-, C- and D-subgenome copies together with an additional copy located on chromosome 2A, consistent with duplication and/or relocation after polyploidization. Similarly, *FaAP2-61* retained four overall copies, although one copy was located on chromosome 5A instead of the expected D-group chromosome, suggesting non-canonical chromosomal placement through structural rearrangement or homeologous exchange.

Other *loci* exhibited partial homeolog retention. On chromosome 2 of *F. vesca*, *FaAP2-47* was represented by only two copies (subgenomes A and C), whereas *FaAP2-48* and *FaAP2-49* retained three copies (A, B and C), suggesting recurrent absence of the D homeolog within this ancestral chromosomal region. Likewise, *FaAP2-13*, located on chromosome 3, retained only the B and D copies, implying stronger fractionation and possible subgenome-biased retention.

More extensive fractionation was observed for *loci* located in chromosome 5. *FaAP2-42* and *FaAP2-43* retained the A-, C- and D-homeologs but lacked the B copy, whereas *FaAP2-65* retained A-, B- and D-homeologs with apparent loss of the C copy. *FaAP2-52* displayed a pattern similar to *FaAP2-42* and *FaAP2-43*, while *FaAP2-51* was represented exclusively by a single B-subgenome copy, constituting one of the clearest examples of homeolog fractionation detected in this study. In contrast, *FaAP2-50* retained four copies overall but exhibited a non-canonical distribution, with an additional copy located on chromosome 7C, suggesting ectopic relocation associated with structural genome rearrangement.

Similar patterns were also detected on chromosome 6. Whereas *FaAP2-14* retained three homeologs (A, B and D), both *FaAP2-11* and *FaAP2-45* were detected exclusively within subgenome B, again indicating extensive fractionation and preferential retention of specific homeologs. Collectively, these observations indicate that *AP2/ERF loci* have not followed a uniform evolutionary trajectory during octoploid genome evolution but instead display multiple retention states ranging from complete four-homeolog conservation to extreme single-copy retention, together with several examples of ectopic chromosomal relocation.

These structural configurations have important implications for the interpretation of gene expression and promoter analyses. Variation in homeolog copy number may influence apparent transcript abundance through gene dosage and presence/absence effects, whereas relocated copies may experience altered chromatin environments and regulatory landscapes that may promote transcriptional divergence. Consequently, promoter *cis*-element composition and expression behaviour should be interpreted within the genomic context of each locus, considering whether individual genes belong to complete homeolog sets, fractionated loci or relocated duplicates, as each evolutionary state may provide distinct opportunities for regulatory divergence, subfunctionalization or neofunctionalization.

Finally, integration of the synteny analysis with the stress-responsive expression data revealed evidence of differential regulatory behaviour among retained homeologs. Whereas drought stress was generally associated with transcriptional induction of *AP2/ERF* genes, salinity responses appeared more heterogeneous, with different homeologs showing either induction or repression under the same stress condition (Figure 2). Although synteny alone cannot explain these transcriptional patterns, the combined structural and expression analyses support the hypothesis that progressive homeolog divergence has contributed to the functional specialization of AP2/ERF family members during adaptation to different abiotic stresses.

### 2.4. Cis-Regulatory Architecture of Drought- and Salinity-Responsive FaAP2/ERF Promoters

**Figure 3:**
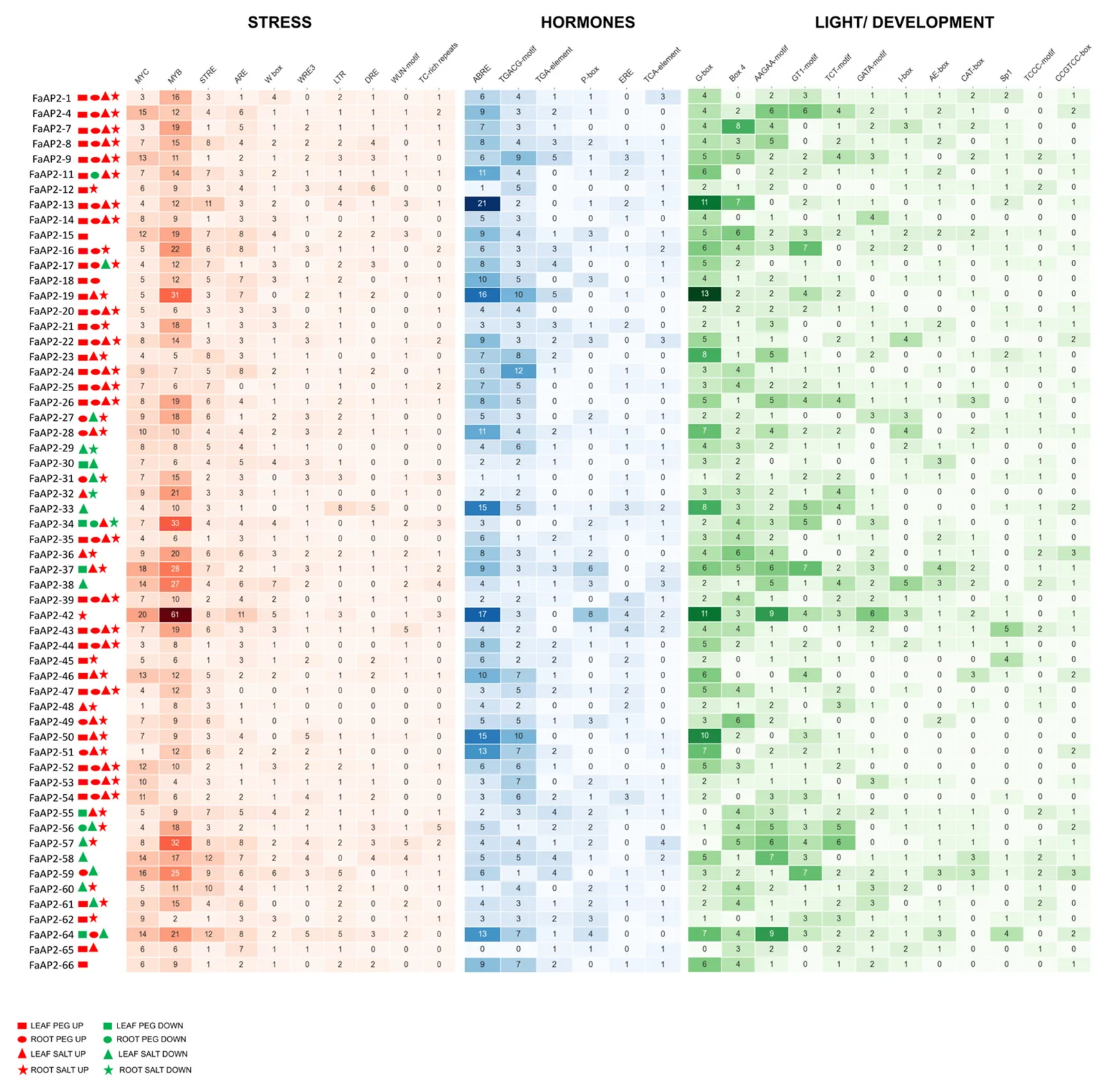
*Cis*-regulatory elements identified in the 2-kb upstream promoter regions of drought- and salinity-responsive *FaAP2/ERF* genes. Heatmap showing the abundance of stress-related (red), hormone-responsive (blue), and light/development-related (green) *cis*-elements in individual promoters. Symbols on the left indicate up- (red) and downregulated (green) genes in leaves and roots under PEG and salt treatments.

Analysis of the 2-kb upstream regions of drought- and salinity-responsive *FaAP2/ERF* genes revealed a highly integrated *cis*-regulatory landscape complex comprising stress-, hormone-, and development-related elements (Figure 3). These observations suggest that MYB-, WRKY- and bHLH-associated regulatory pathways may potentially contribute to the stress-responsive transcription of FaAP2/ERF genes. Overall, the promoter architecture is consistent with the potential integration of multiple regulatory signals associated with adaptive responses to osmotic stress.

Among stress-related *cis*-elements, MYB-binding sites were particularly abundant, with several promoters containing between 15 and 33 copies of these motifs. The high prevalence of MYB elements is consistent with the well-established role of MYB transcription factors in drought- and salinity-responsive gene regulation [36,37]. In addition, motifs associated with bHLH-mediated responses (MYC), general stress regulation (STRE), hypoxia responses (ARE), and WRKY-mediated signaling (W-box) were also widely distributed, indicating that multiple transcriptional networks may converge to modulate FaAP2/ERF expression under abiotic stress conditions [38,39].

Concerning hormone-responsive *cis*-elements, ABRE motifs were the most abundant, reaching up to 21 copies in some promoters. These motifs were found either as isolated elements or as multiple copies within the same promoter region. ABREs are recognized by bZIP transcription factors of the AREB/ABF family and constitute central components of ABA-dependent stress signaling. Several promoters also contained ABRE-associated coupling elements, including CE1, CE3 and motif III, which have been reported to enhance ABA responsiveness. The high prevalence of ABRE/ACGT-containing motifs is therefore consistent with the potential involvement of ABA-associated regulatory processes in drought and salinity responses through the SnRK2–AREB/ABF pathway [40,41].

In addition to ABA-responsive elements, a subset of promoters contained DRE/CRT motifs characterized by the A/GCCGAC core sequence, together with MYB-binding sites associated with drought inducibility. The coexistence of these motifs supports the contribution of both ABA-dependent and ABA-independent pathways to *FaAP2/ERF* transcriptional regulation. DRE/CRT elements are targets of DREB/CBF transcription factors and constitute key components of osmotic stress signaling independent of ABA, whereas MYB proteins have been implicated in drought-inducible transcriptional modules [42]. Together, these observations support the contribution of both ABA-dependent and ABA-independent signaling pathways to FaAP2/ERF regulation.

Several promoters also contained GCC-box elements, which have been associated with ERF-mediated and ethylene-responsive transcriptional regulation. Together with the recurrent occurrence of TGACG and CGTCA motifs involved in jasmonate signaling and W-box elements recognized by WRKY transcription factors, these observations suggest potential crosstalk among ethylene-, jasmonate-, and ROS-related pathways. Such interactions have been described as important components of plant adaptive responses to osmotic stress and may contribute to fine-tuning *FaAP2/ERF* expression in response to changing environmental conditions [14,43].

Additional motifs frequently detected included ARE elements associated with anaerobic and general stress responses, LTR elements involved in low-temperature responsiveness, TCA elements related to salicylic acid signaling, and AuxRE motifs implicated in auxin responses. Light-responsive elements, particularly G-box and Box4 motifs, were also abundant, whereas GT1, GATA-box, I-box and AE-box motifs occurred with variable frequencies. The widespread distribution of these motifs suggests that *FaAP2/ERF* expression may be modulated not only by drought and salinity, but also by developmental and environmental cues. In particular, the G-box represents one of the most versatile cis-elements in plants, being recognized by bZIP, bHLH and other transcription factors involved in both development and stress responses.

Collectively, the *cis*-element landscape of FaAP2/ERF promoters is consistent with a model in which transcriptional responses to drought and salinity may involve a combinatorial promoter architecture containing motifs associated with ABA-, jasmonate-, ethylene-, and ROS-related signaling. Variations in motif composition and abundance among promoters may reflect differences in upstream signaling dominance, tissue specificity, and transcription factor availability. From a functional perspective, the promoter composition suggests the existence of partially overlapping regulatory modules, including a core osmotic-stress module characterized by DRE/CRT and MYB elements, and hormone-associated modules involving ABA, jasmonate and ethylene signaling. Accordingly, the present analysis should be regarded as a framework for generating testable regulatory hypotheses rather than definitive evidence of promoter occupancy.

However, the presence of consensus motifs should be interpreted as evidence of potential regulatory capacity rather than direct evidence of transcription factors binding. Therefore, further characterization using position weight matrix-based approaches and experimental validation through promoter-reporter assays, yeast one-hybrid analyses, EMSA, ChIP-qPCR or DAP-seq under drought, salinity and hormone treatments will be necessary to elucidate the regulatory mechanisms governing *FaAP2/ERF* expression.

### 2.5. Analysis of FaAP2/ERF Expression Patterns in Response to Drought and Salinity

To further validate the transcriptomic dataset, the expression profiles of all 58 selected *FaAP2/ERF* genes were analyzed by RT-qPCR. Although the overall agreement between RNA-seq and RT-qPCR supported the reliability of the transcriptomic dataset, discrepancies were detected for a subset of genes. Therefore, the RT-qPCR results were used as the primary dataset for subsequent expression clustering and biological interpretation, owing to their higher sensitivity and gene-specific quantitative accuracy.

**Figure 4:**
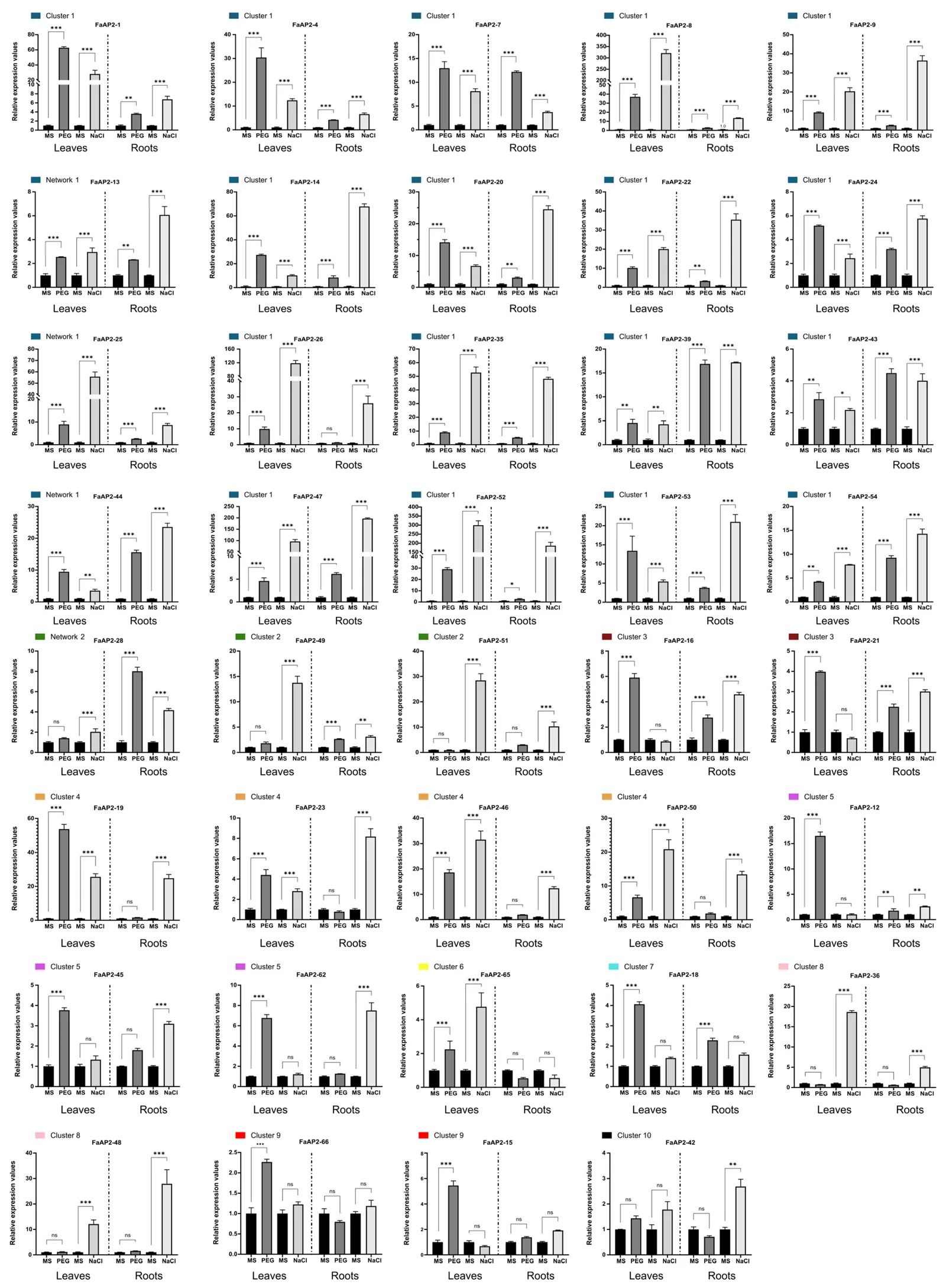
Relative expression profiles of *AP2/ERF* genes belonging to the positively regulated co-expression clusters (upregulated clusters). Gene expression was analyzed in leaves and roots under control (MS), osmotic stress (PEG), and salt stress (NaCl) conditions. Each panel represents an individual gene grouped according to its corresponding co-expression network (Clusters 1–10). Bars represent mean ± SE. Significant differences among treatments are indicated by asterisks (* *p* ≤ 0.5; ** *p* ≤ 0.05; *** *p* ≤ 0.005), whereas ns denotes non-significant differences.

**Figure 5:**
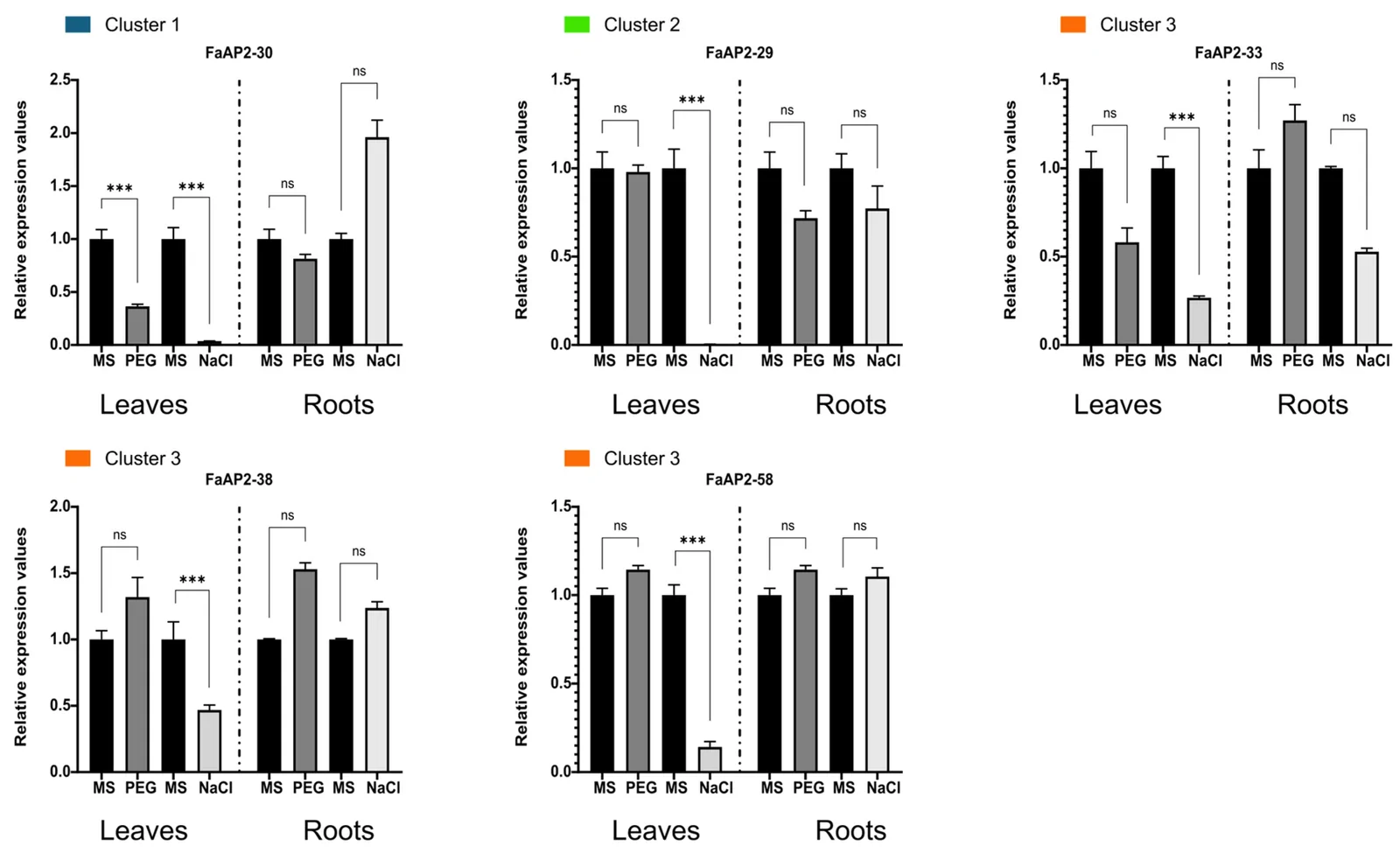
Relative expression profiles of *AP2/ERF* family genes assigned to the negatively regulated co-expression clusters (downregulated clusters). Gene expression was analyzed in leaves and roots under control (MS), osmotic stress (PEG), and salt stress (NaCl) conditions. Each panel represents an individual gene and is grouped according to its corresponding co-expression cluster (Clusters 1–3). Bars represent SE. Significant differences among treatments are indicated by asterisks (*** *p* ≤ 0.005)., whereas ns denotes non-significant differences.

**Figure 6:**
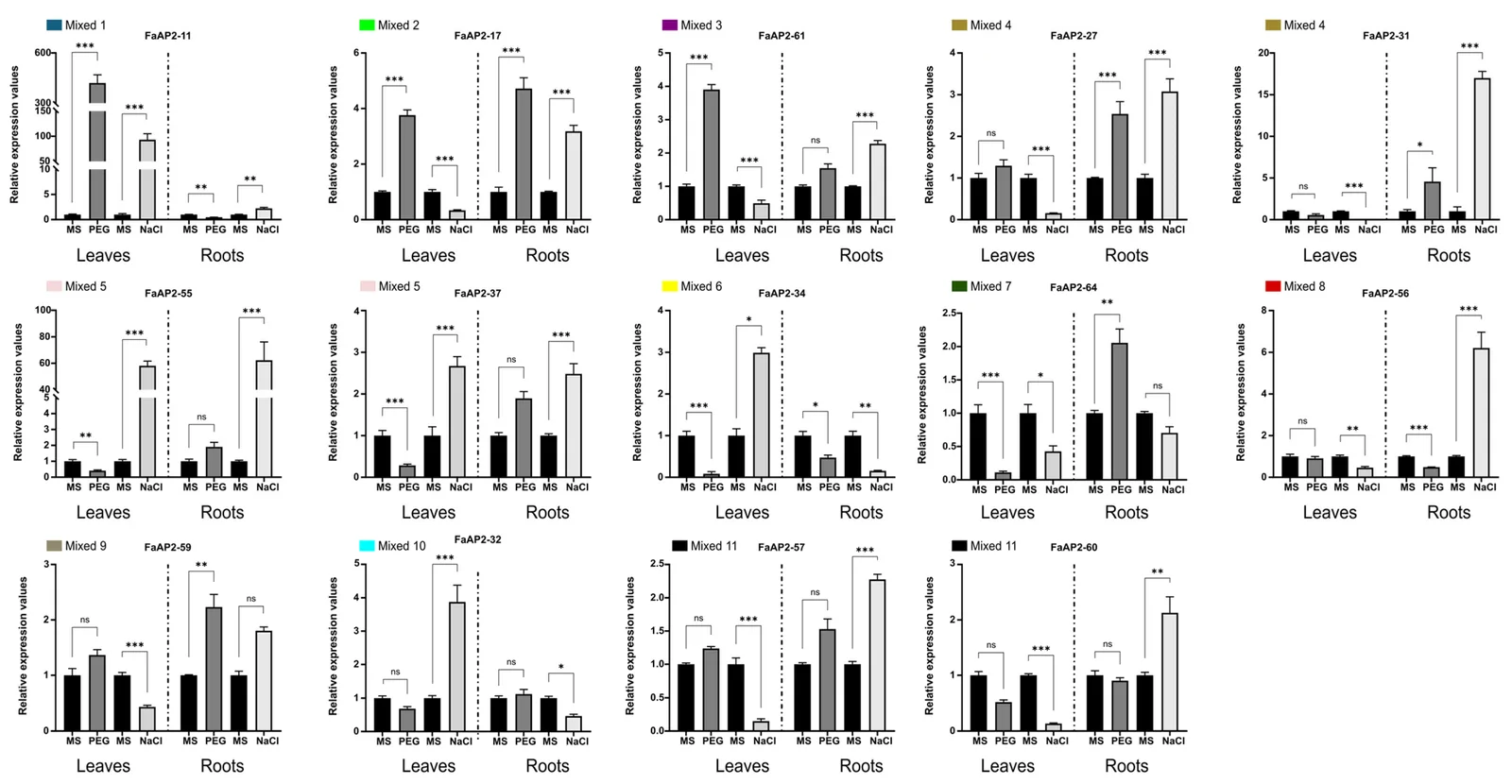
Relative expression profiles of *AP2/ERF* family genes assigned to mixed co-expression clusters (mixed clusters). Gene expression was analyzed in leaves and roots under control (MS), osmotic stress (PEG), and salt stress (NaCl) conditions. Each panel represents an individual gene and is grouped according to its corresponding mixed cluster (Mixed 1–11). Bars represent mean ± SE. Significant differences among treatments are indicated by asterisks (* *p* ≤ 0.5; ** *p* ≤ 0.05; *** *p* ≤ 0.005), whereas ns denotes non-significant differences.

The expression profiles of the selected *AP2/ERF* genes were complex and could be grouped into distinct expression clusters according to their tissue- and stress-specific induction or repression patterns (Figure 4, Figure 5 and Figure 6). The main expression characteristics defining each cluster are summarized in Supplementary Table S3. The presence of several FaAP2/ERF transcription factors within the same expression cluster may reflect partial functional redundancy, shared upstream regulatory mechanisms and/or combinatorial regulation mediated by common cis-regulatory elements and protein–protein interactions. These expression clusters therefore provide a useful framework for integrating promoter architecture with tissue- and stress-dependent transcriptional behavior.

Most of the selected *FaAP2/ERF* genes were assigned to upregulated clusters. Cluster 1 represented the broadest stress-responsive pattern and included genes upregulated in leaves and roots under both drought-like osmotic stress and salt stress. This cluster comprised 20 genes, suggesting the existence of a core group of FaAP2/ERF regulators broadly responsive to osmotic and salt-related stress conditions. Other upregulated clusters displayed more restricted patterns, including preferential induction in roots, leaves or specific combinations of drought and salt stress. These profiles suggest that, in addition to a general stress-responsive component, *FaAP2/ERF* regulation also involves tissue-specific and stress-specific transcriptional modules.

A smaller number of genes were assigned to downregulated clusters. These genes showed repression either in both drought- and salt-stressed leaves or specifically under salinity conditions. Although less represented than the upregulated clusters, these repressed genes may also contribute to the stress response by attenuating developmental, metabolic or regulatory programmes that are incompatible with acclimation. Therefore, repression of selected *FaAP2/ERF* genes should not be interpreted as a passive response, but rather as a potential component of transcriptional reprogramming under stress.

A third group of genes showed mixed expression profiles, with induction or repression depending on tissue type and stress condition. This group is particularly informative because it suggests that some *FaAP2/ERF* genes may integrate stress and tissue-specific information rather than responding uniformly to a given environmental cue.

Overall, these results indicate that most members of the selected *FaAP2/ERF* set display partially overlapping but non-identical expression profiles in response to drought-like osmotic stress and salinity. This pattern is consistent with the central role of AP2/ERF transcription factors in coordinating abiotic-stress responses through both hormone-dependent and hormone-independent regulatory pathways [9,10]. Several non-mutually exclusive mechanisms may account for this expression behavior.

Our results indicate that most members of the FaAP2/ERF family show partially overlapping expression profiles in response to the two abiotic stresses analyzed, namely drought and salinity, while being assigned to distinct expression networks (Figure 4, Figure 5 and Figure 6). This pattern is consistent with the central role of AP2/ERF transcription factors in coordinating abiotic-stress responses through both hormone-dependent and hormone-independent regulatory pathways [13,14]. Several non-mutually exclusive mechanisms may account for this expression behavior. (A) partial functional redundancy among several ERFs may underline their membership in a common stress-responsive module, allowing this module to remain active even if one of its components is impaired, for example due to mutations in coding sequences or promoter regions. Such redundancy is particularly plausible in polyploid genomes, where duplicated or homeologous genes can contribute to robustness stress-response while progressively diverging in expression patterns or regulatory functions [34,44,45]; (B) subfunctionalization may have occurred, whereby different ERFs respond to the same general stimuli but play distinct roles within specific temporal windows or according to stress intensity, with some factors responding preferentially to mild stress and others to severe stress; (C) cell-type specificity within leaves may contribute to these differences, as individual genes may be expressed in distinct cellular contexts. This interpretation is supported by recent single-cell transcriptomic analyses showing that drought responses in leaves are spatially and cell-type resolved rather than uniformly activated across the whole organ [46]; (D) promoter architecture is likely to be relevant, since members of the same TF family may not recognize or integrate exactly the same *cis*-regulatory sequence code. In this regard, the combinatorial distribution, spacing and local context of *cis*-regulatory elements can strongly influence spatiotemporal gene expression and environmental responsiveness [47,48]; (E) recruitment of different cofactors may result in distinct transcriptional outputs, as the same ERF can potentially act as an activator or a repressor depending on its interaction partners, including other transcription factors, Mediator components or chromatin-associated regulators. The Mediator complex provides a mechanistic link between sequence-specific transcription factors and the basal transcriptional machinery, thereby contributing to the activation or repression of target genes in response to environmental cues [49]. In this context, hierarchical regulatory clusters and transcription factor–transcription factor interactions may represent an important component of the regulatory wiring underlying drought and salinity responses [13,14].

Finally, combinatorial regulation at the same promoter is also plausible, as two or more ERFs may co-occupy regulatory regions of their target genes, either cooperatively or competitively. Taken together, these observations support a hierarchical model of FaAP2/ERF regulation in which partially redundant transcription factors integrate promoter architecture, tissue context, stress intensity and cofactor availability to generate distinct transcriptional outputs during drought and salinity responses. Their transcriptional outputs may depend on duplicated gene divergence, promoter *cis*-regulatory architecture, cell-type context, stress intensity and recruitment of specific transcriptional cofactors [9,10,50,51].

### 2.6. Functional Characterization of FaAP2-11

Based on the combined RNA-seq and RT-qPCR analyses, *FaAP2-11* was selected for further functional characterization because it displayed a robust, reproducible and highly abundant stress-responsive expression profile. *FaAP2-11* belonged to a mixed-response expression cluster, being induced in drought-stressed leaves, salt-stressed leaves and salt-stressed roots, while being repressed in drought-stressed roots. Despite these contrasting tissue-specific responses, *FaAP2-11* maintained high transcript abundance across all stress conditions analyzed, making it one of the most strongly expressed *AP2/ERF* genes identified. Its marked induction in leaf tissues under both drought and salinity, together with its contrasting expression pattern between aerial and root tissues, suggests that *FaAP2-11* may contribute to the coordination of tissue-specific regulatory programmes involved in abiotic stress acclimation.

#### 2.6.1. Spatial Expression Profile of *FaAP2-11*

**Figure 7:**
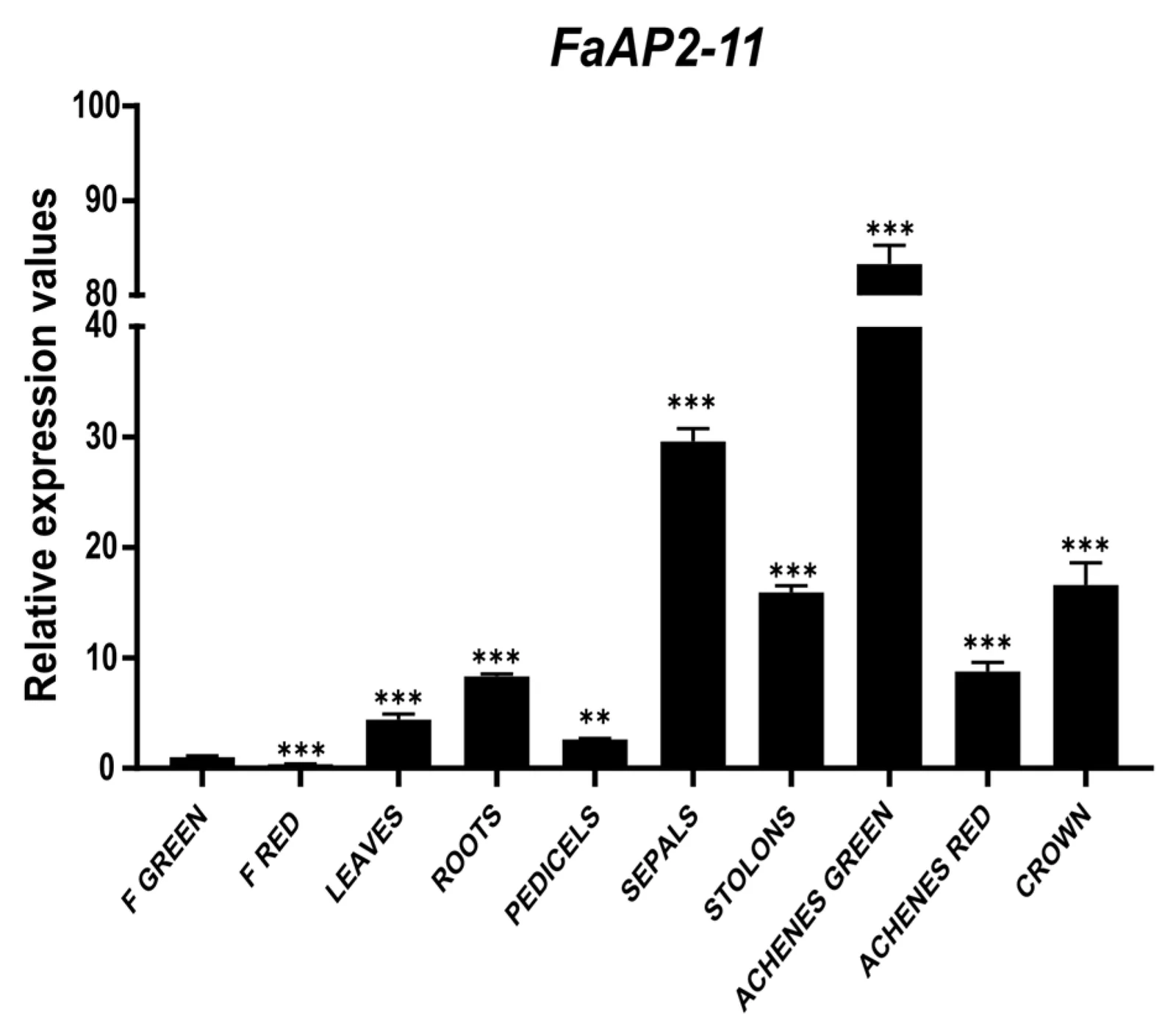
Relative expression profile of *FaAP2-11* in different tissues and developmental stages of *Fragaria × ananassa*. Transcript levels were determined by RT-qPCR and normalized to green fruit (F GREEN), which was set to 1. Bars represent the mean ± SE of three biological replicates. Asterisks indicate significant differences relative to F GREEN (** *p* < 0.05, *** *p* < 0.005). Tissue abbreviations: F GREEN, green fruit; F RED, red fruit.

Expression analyses of *FaAP2-11* were performed in different strawberry tissues, including crown, runners, sepals, pedicels, roots, leaves, green-fruit receptacles, red ripe-fruit receptacles, green-fruit achenes and red-fruit achenes (Figure 7). *FaAP2-11* transcripts accumulated predominantly in immature green-fruit achenes, followed by sepals, crown, runners, red-fruit achenes, roots and pedicels. In contrast, transcript abundance remained consistently low in receptacle tissues at both the green and red-ripe stages, indicating that *FaAP2-11* expression is preferentially associated with reproductive structures other than the fleshy receptacle.

At first glance, the preferential accumulation of *FaAP2-11* transcripts in both immature achenes and sepals appears unexpected because these organs differ markedly in their developmental origin and biological function. Achenes represent the true botanical fruits containing the developing seeds, whereas sepals constitute floral-derived protective organs. Therefore, the common expression pattern observed in these tissues is unlikely to reflect organ identity itself. Rather, it suggests that *FaAP2-11* may respond to physiological processes shared by both tissues during fruit development.

Support for this interpretation comes from studies in several angiosperm species showing that persistent green sepals or calyces remain physiologically active after pollination and contribute directly to reproductive performance. Rather than acting as passive floral remnants, these organs participate in maintaining favorable microenvironmental conditions around developing fruits and seeds, contribute photosynthetically derived assimilates and influence reproductive fitness. Such functions have been experimentally demonstrated in *Helleborus foetidus*, *Paris polyphylla*, *Spinacia oleracea* and *Salvia miltiorrhiza*, where persistent sepals or calyces affect seed development, fruit growth, carbon metabolism and female reproductive success [50,51,52,53]. Although a direct regulatory relationship between achenes and sepals has not been demonstrated in strawberry, these observations support the existence of partially overlapping physiological programmes operating in both reproductive and floral-derived protective tissues.

In strawberry, developing achenes constitute the principal regulatory centers controlling early fruit development. Auxin accumulates predominantly within achenes and orchestrates the initiation of receptacle growth following fertilization [52,54]. As seed development progresses, additional physiological processes become activated, including embryo development, reserve accumulation, ABA-dependent maturation, acquisition of desiccation tolerance and activation of protective mechanisms involving LEA proteins, dehydrins, heat-shock proteins, antioxidant systems and redox homeostasis. ABA assumes a particularly important regulatory role during seed maturation by promoting dehydration-associated protection and coordinating maturation programmes. In addition, because strawberry is a non-climacteric fruit, ABA also contributes to fruit ripening through extensive interactions with auxin and other hormonal signaling pathways [55].

Taken together, these observations suggest that the high expression of *FaAP2-11* in immature achenes is fully consistent with a regulatory role associated with hormone-mediated developmental programmes, progressive acquisition of stress tolerance and protection against dehydration and oxidative stress during seed maturation. An additional point of interest is that some strawberry genes can be expressed in both fruit and floral organs. For instance, *FaDOF2* has been described as highly expressed in ripe fruit receptacles and also in petals, and its expression correlates with the accumulation of eugenol, a phenylpropanoid volatile, in both tissues. This observation supports the idea that certain floral and fruit metabolic modules may partially overlap during strawberry reproductive development [56].

#### 2.6.2. Induction of *FaAP2-11* by Pro-Oxidant and Hormone-Related Treatments

Drought and salinity rapidly perturb redox homeostasis in plants, leading to the accumulation of reactive oxygen species (ROS), including superoxide anion (O_2_^•−^), hydrogen peroxide (H_2_O_2_) and hydroxyl radicals (^•^OH). At controlled levels, ROS act as early signaling intermediates that activate acclimation pathways, whereas their over accumulation causes oxidative damage to lipids, proteins, nucleic acids and the photosynthetic machinery [57]. In this context, AP2/ERF transcription factors, particularly DREB- and ERF-type regulators, are widely recognized as central components of abiotic-stress signaling networks, integrating osmotic, hormonal and redox-related cues with downstream transcriptional programmes associated with cellular protection [13,28].

**Figure 8:**
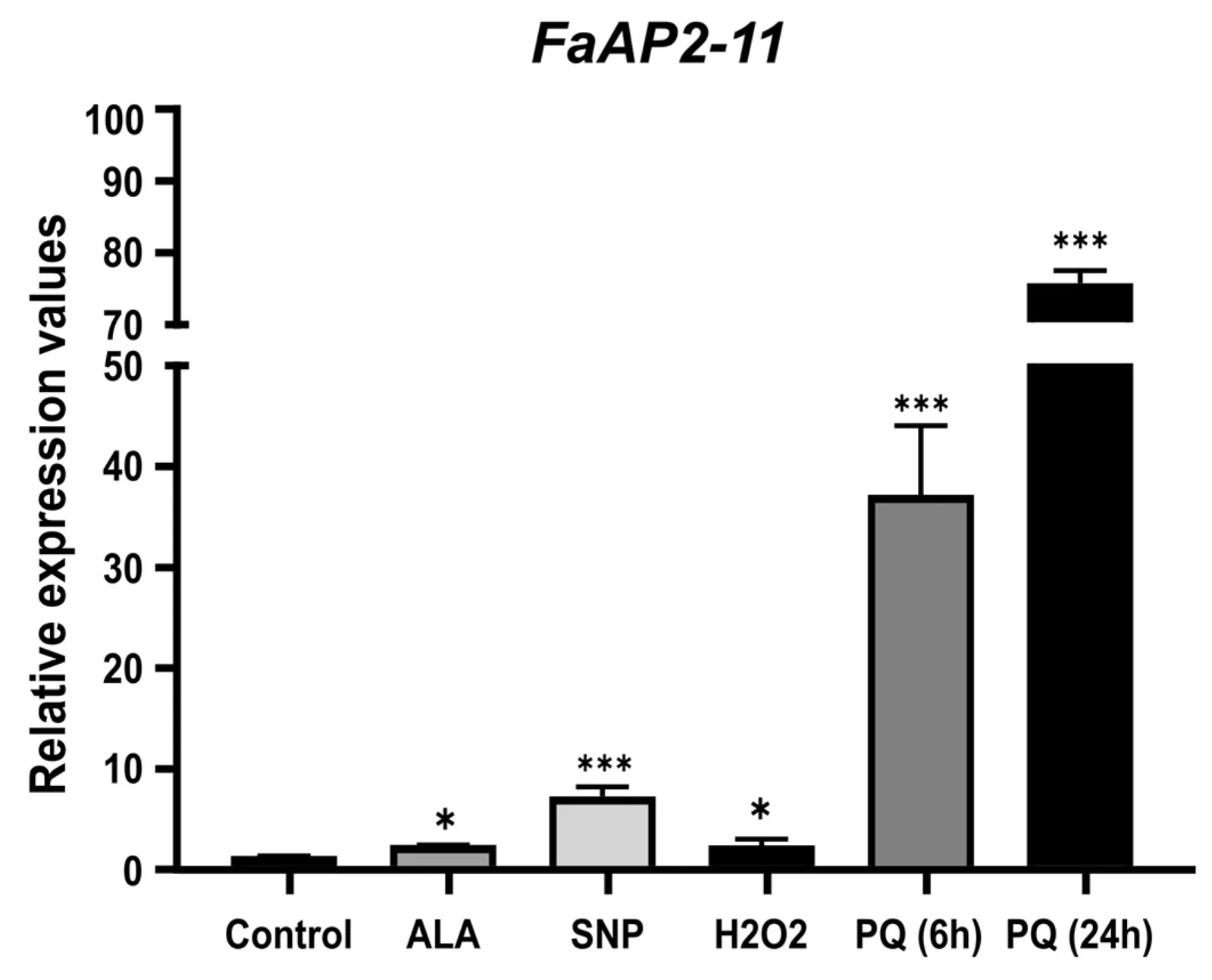
Relative expression of *FaAP2-11* in leaves of *Fragaria × ananassa* cv. Chandler following treatments with oxidative stress-related compounds. Transcript levels were measured by RT-qPCR after treatment with 5-aminolevulinic acid (ALA), sodium nitroprusside (SNP), hydrogen peroxide (H_2_O_2_), and paraquat (PQ) for 6 h and 24 h. Expression levels were normalized to the untreated control (set to 1). Data represents the mean ± SE of three biological replicates. Significant differences relative to the control are indicated by asterisks (* *p* < 0.5, *** *p* < 0.005).

To determine whether *FaAP2-11* is responsive to oxidative-stress-related signals, strawberry leaves were treated with ROS- or redox-associated compounds, including paraquat (PQ), hydrogen peroxide (H_2_O_2_), 5-aminolevulinic acid (ALA) and sodium nitroprusside (SNP). Paraquat, also known as methyl viologen, is a redox-active herbicide that interrupts the photosynthetic electron-transport chain, promoting superoxide formation and inhibiting NADPH production in the chloroplast [58], whereas SNP acts primarily as a nitric-oxide donor. *FaAP2-11* transcript levels increased in response to all treatments (Figure 8), although the magnitude of induction differed markedly among compounds. The strongest induction was observed after PQ treatment, which increased *FaAP2-11* expression by 36.9-fold, followed by SNP treatment, which resulted in a 6.9-fold increase. In contrast, both ALA and H_2_O_2_ had only a limited effect on *FaAP2-11* transcript accumulation, with an approximately 2.1-fold induction in each case.

These results indicate that *FaAP2-11* expression is strongly influenced by signaling molecules or redox perturbations associated with oxidative stress, such as those triggered under drought or salinity conditions. Moreover, the induction observed after SNP treatment suggests that *FaAP2-11* may also be connected with nitric-oxide-dependent or nitro-oxidative signaling pathways [59]. The strong induction of *FaAP2-11* by paraquat, together with its weaker responsiveness to exogenous H_2_O_2_ and ALA, suggests that this transcription factor may be preferentially activated by chloroplast-derived photooxidative signaling, most likely involving superoxide-derived redox perturbation and secondary H_2_O_2_ signaling, with an additional contribution of nitro-oxidative cues under SNP treatment.

Stress acclimation depends to a large extent on the coordinated induction and/or activation of antioxidant systems, including superoxide dismutase, catalases, ascorbate peroxidases, glutathione peroxidases, glutathione reductases and peroxiredoxins, which restrict excessive ROS accumulation and contribute to the re-establishment of redox balance [57]. In strawberry, this framework is supported by genome-wide studies showing that AP2/ERF genes are broadly represented in *Fragaria vesca*, including drought-responsive *FvDREB* members such as *FvDREB6* and *FvDREB8*, as well as recent characterizations of ROS-scavenging gene families, including SOD and APX, in *Fragaria* [33,34,60,61]. Together, these findings suggest that *FaAP2-11* may participate in a redox-sensitive transcriptional module that links drought- and salinity-induced oxidative stress with protective antioxidant and stress-acclimation responses.

**Figure 9:**
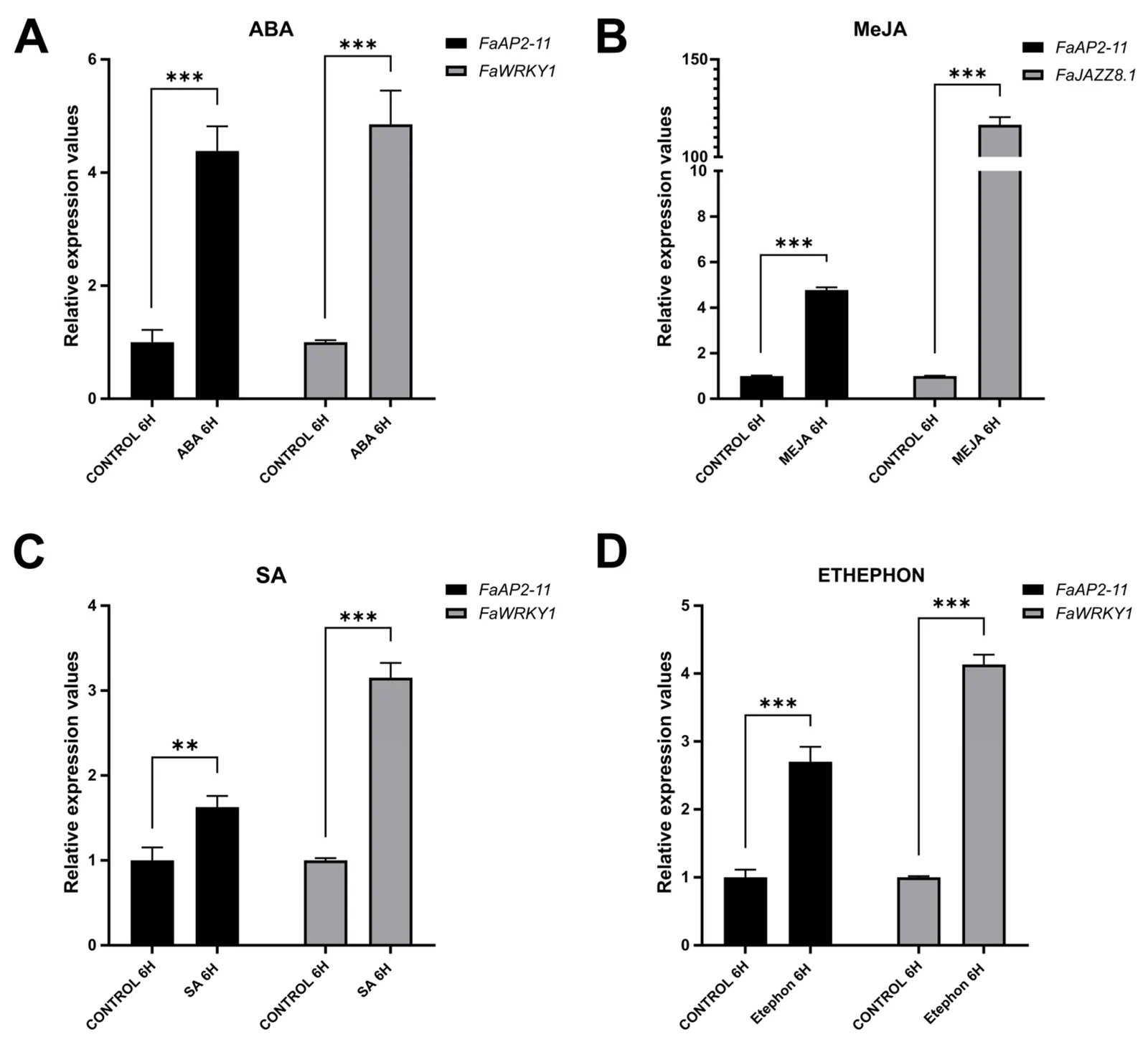
Relative expression of *FaAP2-11* in leaves of *Fragaria × ananassa* cv. Chandler following treatment with different stress-related phytohormones. Transcript levels were determined by RT-qPCR after 6 h of treatment with (**A**) abscisic acid (ABA), (**B**) methyl jasmonate (MeJA), (**C**) salicylic acid (SA), and (**D**) ethephon. Expression values were normalized to the corresponding untreated controls, which were set to 1. The expression of hormone-responsive marker genes, *FaWRKY1* (ABA, SA, and ethephon treatments) and *FaJAZ8.1* (MeJA treatment), was analyzed to verify treatment efficacy. Bars represent the mean ± SE of three biological replicates. Significant differences relative to the corresponding control are indicated by asterisks (** *p* < 0.05, *** *p* < 0.005).

Hormonal signaling constitutes a major component of plant adaptation to drought and salinity. To investigate whether *FaAP2-11* responds to stress-associated phytohormones, leaves of *Fragaria × ananassa* cv. Chandler were treated with ABA, methyl jasmonate (MeJA), salicylic acid (SA), and ethephon, and transcript accumulation was analyzed by RT-qPCR (Figure 9). The induction of the marker genes *FaWRKY1* for ABA, SA and ethephon treatments and *FaJAZ8.1* for MeJA treatment confirmed the effectiveness of the corresponding treatments [62]. *FaAP2-11* expression increased significantly after all hormone treatments, although the magnitude of induction varied among compounds. ABA and MeJA elicited the strongest responses, increasing transcript levels by 4.4- and 4.8-fold, respectively, whereas ethephon and SA produced more moderate inductions of 2.7- and 1.6-fold.

AP2/ERF proteins are widely recognized as central regulators of abiotic-stress responses and frequently act as convergence points for multiple signaling pathways, including ABA-, ethylene- and jasmonate-dependent clusters [13,28]. ABA is considered the principal hormonal regulator of drought and salinity adaptation, coordinating stomatal closure, osmotic adjustment, ROS detoxification and stress-responsive gene expression, whereas jasmonates, ethylene and salicylic acid provide additional regulatory layers involved in antioxidant defense and stress acclimation [63]. The pronounced responsiveness of *FaAP2-11* to ABA therefore suggests that this transcription factor may participate in ABA-mediated adaptive pathways activated under water deficit and salt stress. Likewise, MeJA has been shown to contribute to abiotic stress tolerance through extensive crosstalk with ROS signaling and antioxidant metabolism [63,64], indicating that the strong induction of *FaAP2-11* by MeJA may reflect its involvement in jasmonate-dependent stress-acclimation pathways. In contrast, the weaker induction observed after ethephon, and SA treatments suggest that ethylene- and salicylic acid-dependent pathways may exert a secondary or modulatory effect on *FaAP2-11* expression.

Interestingly, the responsiveness of *FaAP2-11* to multiple phytohormones parallels its marked induction by paraquat and sodium nitroprusside observed in the oxidative stress experiments. Reactive oxygen species and nitric oxide are key signaling molecules generated during drought and salinity stress, and AP2/ERF transcription factors have been proposed to integrate hormonal and redox-related signals to coordinate downstream protective responses [13,28]. Therefore, the combined responsiveness of *FaAP2-11* to ABA, MeJA, ethylene, SA and oxidative stress-related signals suggest that this transcription factor may function as part of a signaling network linking hormonal and redox cues with stress acclimation processes. Further studies aimed at identifying its downstream targets will be necessary to clarify its precise regulatory role.

#### 2.6.3. Heterologous Overexpression of *FaAP2-11* in *Nicotiana benthamiana* and Physiological Characterization

**Figure 10:**
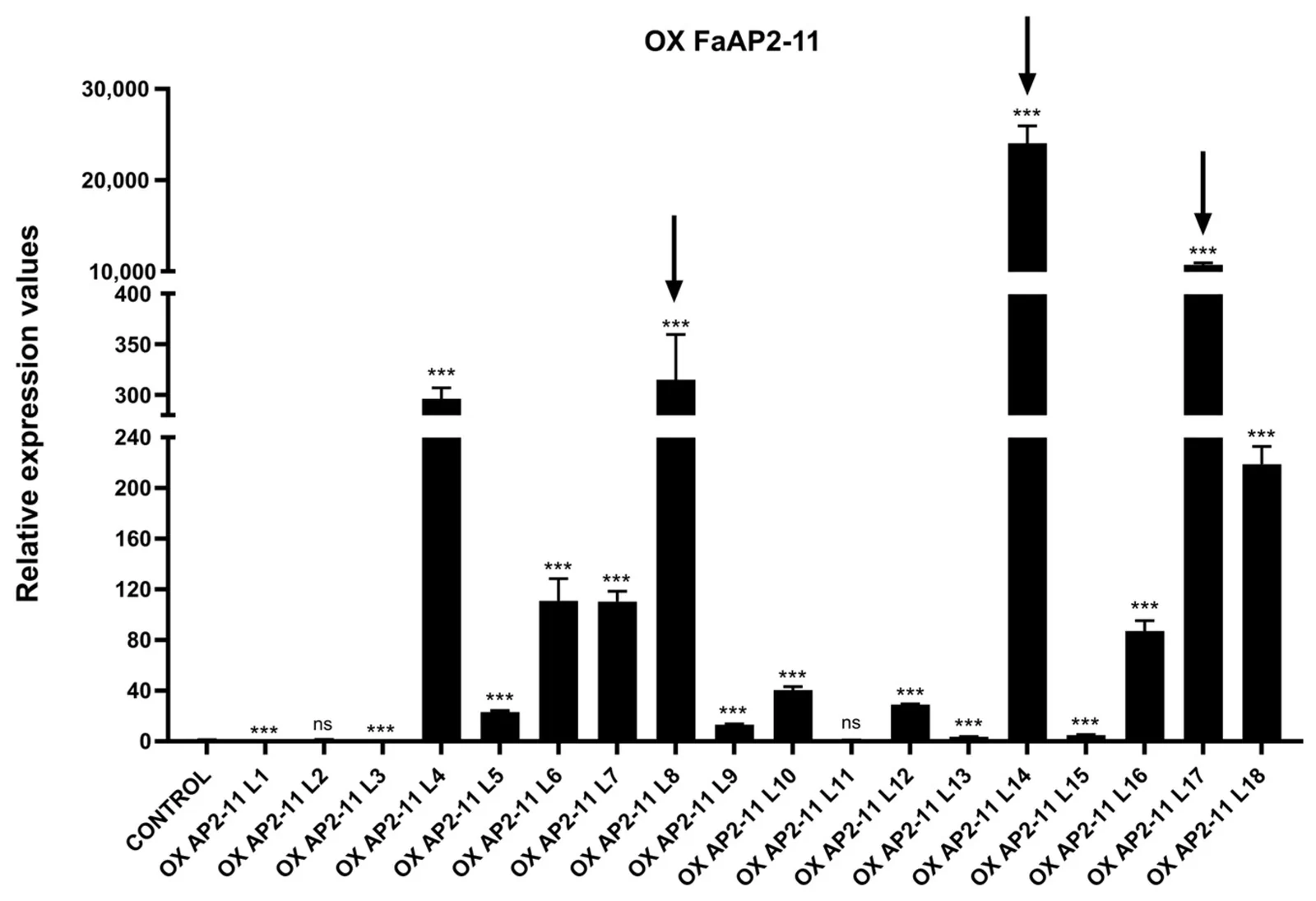
Relative expression of *FaAP2-11* in independent *Nicotiana benthamiana* overexpression lines. Transcript levels were quantified by RT-qPCR and normalized to the empty-vector control (set to 1). Arrows indicate the three lines (*FaAP2-11*-OX L8, L14 and L17) selected for further physiological analyses. Data represents the mean ± SE of three biological replicates. Asterisks indicate significant differences relative to the empty-vector control (*** *p* < 0.005); ns, non-significant.

Heterologous expression systems in plant species such as *Nicotiana benthamiana* and *Arabidopsis thaliana* have been widely used as suitable approaches for the functional characterization of plant transcription factors [9,65]. To investigate the physiological function of *FaAP2-11*, the gene was heterologously overexpressed in *Nicotiana benthamiana*. Several independent transgenic lines were generated, and the three lines exhibiting the highest transcript accumulation (L8, L14 and L17) were selected for physiological characterization (Figure 10). Gas-exchange analyses were subsequently performed using an infrared gas analyzer (IRGA) to evaluate photosynthetic performance, stomatal behavior and water-use-related traits under drought and salinity stress.

These selected lines were subjected to gas-exchange analyses using an infrared gas analyzer (IRGA). The physiological parameters measured included net CO_2_ assimilation rate (A), intercellular CO_2_ concentration (Ci), leaf vapour pressure deficit (VPDleaf), stomatal conductance (gs) and transpiration rate (E). This experimental approach was aimed at determining whether *FaAP2-11* overexpression modifies photosynthetic performance, stomatal behavior and water-use-related physiological responses under stress conditions.

**Figure 11:**
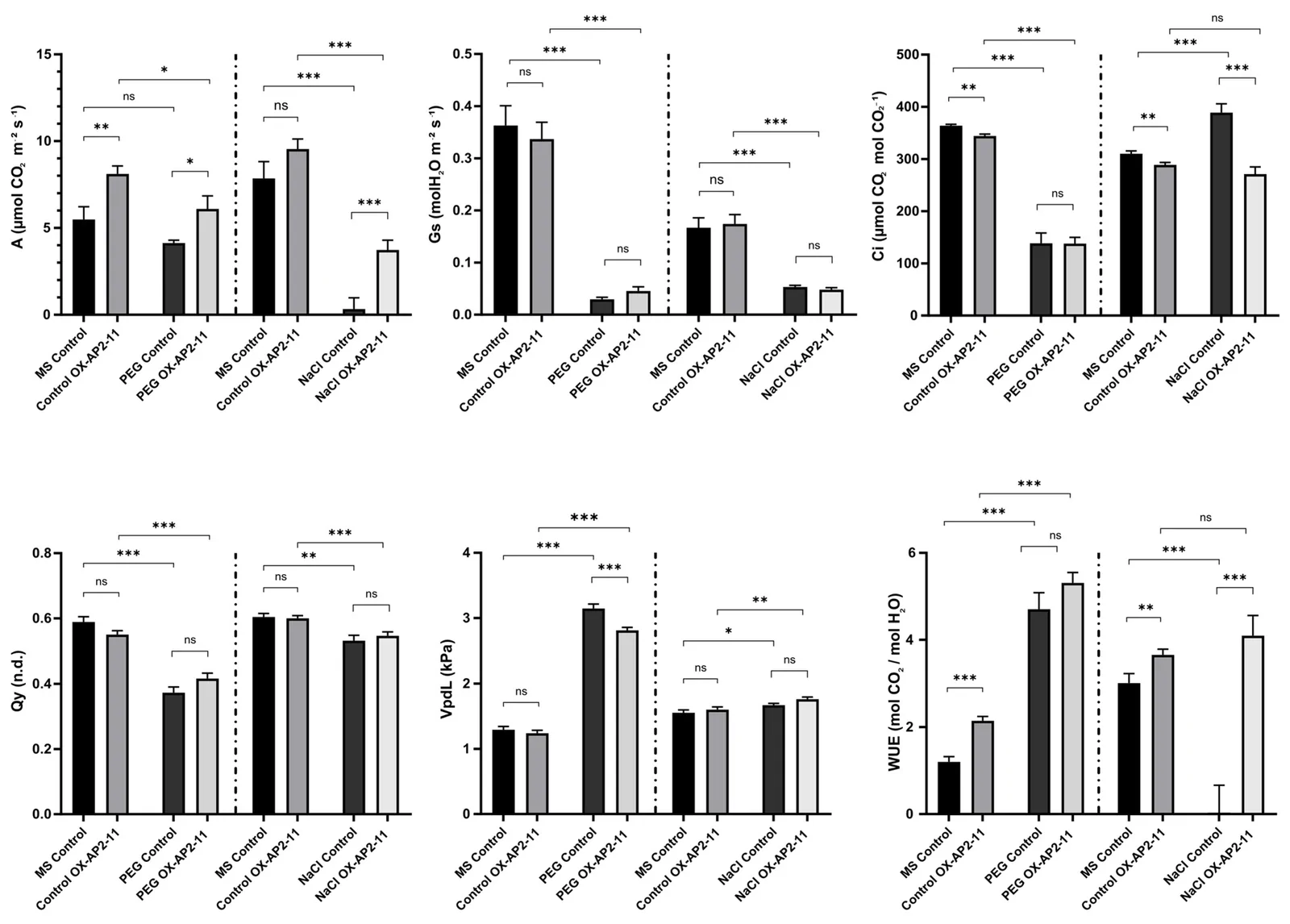
Gas-exchange and water-use-related parameters of wild-type (WT) and *FaAP2-11* overexpression (*FaAP2-11-OX*) *Nicotiana benthamiana* plants under control conditions and after osmotic (PEG) and salt (NaCl) stress treatments. Measurements obtained using an infrared gas analyzer (IRGA) included net CO_2_ assimilation rate (A), stomatal conductance (gs), intercellular CO_2_ concentration (Ci), maximum quantum yield of PSII (Qy), leaf vapor pressure deficit (VPDleaf), and intrinsic water-use efficiency (WUE). Bars represent the mean ± SE of three biological replicates. Significant differences are indicated by asterisks (* *p* < 0.5, ** *p* < 0.05, *** *p* < 0.005); ns, non-significant.

The analysis of physiological variables revealed marked differences depending on both the treatment and the type of stress imposed. In all genotypes, drought and salinity reduced stomatal conductance, transpiration and photosynthetic performance. However, *FaAP2-11*-transformed plants maintained significantly higher net CO_2_ assimilation rates under stress conditions than empty-vector control plants. In addition, *FaAP2-11*-overexpressing plants showed lower intercellular CO_2_ concentration, which was associated with increased intrinsic water-use efficiency, particularly under salinity stress. Another relevant difference was observed in leaf vapour pressure deficit, with transformed plants displaying significantly lower VPDleaf values than control plants (Figure 11).

High VPDleaf values are generally associated with reduced hydraulic conductivity and stomatal closure, although a strong vapour-pressure gradient between the leaf and the surrounding atmosphere may also be linked to increased transpiration rates [66]. In this context, *FaAP2-11*-overexpressing plants appeared to exhibit improved hydraulic regulation under stress conditions. This response is consistent with the reported involvement of AP2/ERF transcription factors in stress priming and in activation of protective mechanisms against osmotic stress [9,13,28]. However, while these leaf-level gas-exchange measurements demonstrate an optimized balance between carbon gain and water loss, whether this translates into improved whole-plant performance remains to be confirmed. The lack of additional phenotypic and physiological evaluations, such as biomass accumulation, leaf water status, growth recovery after stress, or ion accumulation under salinity, represents a limitation of the present study. Future work integrating these traits will be essential to establish if the observed photosynthetic advantages ultimately lead to superior plant growth and stress tolerance.

#### 2.6.4. RNA-Seq Analysis of FaAP2-11-Overexpressing *Nicotiana benthamiana* Lines

Transcriptomic analysis of leaves from *FaAP2-11*-overexpressing *Nicotiana benthamiana* plants revealed extensive transcriptional changes following ectopic *FaAP2-11* expression. The initial differential expression analysis performed by Novogene (adjusted *p* < 0.05) identified 3.494 differentially expressed genes (DEGs), comprising 1.615 upregulated and 1.879 downregulated genes. GO enrichment analysis of this complete DEG set indicated that the most significantly affected biological processes were related to photosynthesis, glucan and polysaccharide metabolism, and responses to environmental stimuli, whereas enriched cellular components were predominantly associated with photosynthetic structures. Likewise, molecular function categories were enriched in glycosyltransferase, hydrolase and oxidoreductase activities, as well as iron ion, calcium ion and tetrapyrrole binding. Consistent with these results, KEGG analysis identified photosynthesis, photosynthetic antenna proteins, starch and sucrose metabolism, phenylpropanoid biosynthesis and other secondary metabolic pathways among the most significantly enriched pathways (Supplementary Figure S2).

After the initial differential expression analysis, a subset of robustly regulated genes was selected for functional interpretation using more stringent filtering criteria: adjusted *p*-value ≤ 0.05, FPKM ≥ 5, and a fold-change cutoff of ≥3 for upregulated genes or ≤1/3 for downregulated genes. Upregulated and downregulated genes were analyzed separately. This filtering strategy identified 314 differentially expressed genes, of which 67 were upregulated and 247 were downregulated.

To independently validate the transcriptomic data, the expression of selected differentially expressed genes representing both upregulated and downregulated transcripts was assessed by RT-qPCR. The RT-qPCR results showed expression trends highly consistent with those obtained by RNA-seq, supporting the reliability of the transcriptomic dataset (Supplementary Figure S3).

To facilitate biological interpretation, the filtered DEG dataset was organized into six putative functional modules (Table 1) based on gene annotation and curated functional grouping (Supplementary Table S4). This classification should not be interpreted as a formal GO or KEGG enrichment analysis, but rather as an interpretative framework aimed at identifying the major biological processes potentially affected by *FaAP2-11* overexpression. The six functional modules summarized in Table 1 revealed coherent transcriptional trends associated with signal perception and transduction, transcriptional regulation, primary metabolism, cell wall remodeling, redox homeostasis and detoxification, and specialized metabolism. Building on this classification, the relationships among these modules were integrated into a conceptual model that summarizes the principal biological processes underlying the transcriptional reprogramming observed in *FaAP2-11*-overexpressing plants (Table 2). This integrative model should therefore be regarded as a biologically informed synthesis of the curated functional classification rather than as an independent functional enrichment analysis.

The curated functional classification revealed that the transcriptional changes associated with *FaAP2-11* overexpression were not randomly distributed across biological processes but instead converged into six interconnected functional modules. Although discussed individually below, these modules should be viewed as components of an integrated regulatory programme rather than as independent pathways.

**Hormonal reprogramming and stress signaling**. Cluster A comprised genes associated with hormone metabolism, hormone signaling, Ca^2+^-dependent signaling, osmotic/mechanical sensing, circadian regulation and light-related responses. Overall, the expression profile suggests a shift from growth-promoting regulatory programmes towards stress-associated hormonal adjustment. This interpretation is supported by the induction of *NbU74E2/UGT74E2*, which may reduce the active auxin pool through auxin conjugation [67] together with the repression of *NbPATL3/4* and *NbGA3ox*, suggesting reduced auxin transport-related processes and gibberellin biosynthesis [68,69]. These changes are consistent with attenuation of auxin- and gibberellin-dependent growth programmes [70,71,72]. In parallel, the repression of *NbABAH2*, encoding an ABA 8′-hydroxylase involved in ABA catabolism, and *NbPUB19*, a negative regulator of ABA responses, suggest enhanced ABA signaling capacity [73,74]. Additional changes, including the induction of *NbAOS2* and the repression of genes related to brassinosteroid, jasmonate and growth-associated signaling, further support a hormonal rebalancing compatible with stress acclimation [55,75,76,77,78,79]. The downregulation of *NbCML37/38*, *NbCIPK5/6*, *NbOSCA1.2* and *NbLRX3* further suggests attenuation of Ca^2+^-dependent signaling and mechanical/osmotic sensing at the plasma membrane and cell wall [80,81,82,83,84].

**Transcriptional and regulatory reprogramming**. Cluster B comprised genes related to transcriptional and post-transcriptional regulation, chromatin control, RNA processing, protein turnover, programmed cell death, hypersensitive response and senescence-associated pathways. The most pronounced repression in the dataset affected *NbDREB1A* and *NbDREB1D*, two members of the DREB/CBF regulon involved in cold and dehydration responses [8,15,85]. This marked repression, together with the downregulation of *NbHB7*, *NbWRKY40*, *NbZAT5* and other stress-associated regulators, suggests that *FaAP2-11* does not constitutively activate the classical DREB/LEA-type stress programme. Instead, *FaAP2-11* may promote an alternative regulatory state in which master stress regulons and growth-associated transcriptional programmes are attenuated, partially resembling the repressive role described for DDF1/2-related AP2/ERF regulators [86]. The downregulation of *NbFLZ8* and *NbHB7* further supports the hypothesis that *FaAP2-11* may interfere with growth-to-stress regulatory switches involving SnRK1/energy signaling and ABA-associated growth control [87,88,89,90]. In addition, changes in chromatin-associated regulators, including induction of *NbHLS1* and repression of *NbALPL/NbALP1*, suggest that *FaAP2-11*-dependent reprogramming may also involve chromatin-level regulation [91,92,93]. The upregulation of *NbLaRP6C*, *NbESP3*, *NbSCE1* and *NbUTR1* suggests that selected RNA-stability, splicing and protein-protection mechanisms are maintained [94,95,96,97], whereas the broader repression of genes involved in chaperone activity and protein turnover is consistent with an energy-saving strategy under stress-related conditions [98]. Interestingly, the induction of *NbNAC6* and *NbHIR1*, together with the repression of several *NbSAG12* copies, suggests activation of defense- or PCD/HR-associated signaling without progression towards terminal senescence [99,100,101].

**Metabolic adjustment and energy reallocation**. Cluster C, corresponding to primary metabolism reprogramming, redox homeostasis and detoxification, included genes associated with photosynthesis, the Calvin cycle, energy metabolism, sugar metabolism, lipid metabolism, redox balance and selected detoxification-related processes. The repression of photosynthetic genes such as *NbPLAS* and *NbPSAO*, together with the downregulation of genes involved in raffinose biosynthesis, carbohydrate interconversion, phosphate-dependent cellulose production and nucleotide metabolism, indicates reduced investment in energetically costly processes such as photosynthesis, growth and wall expansion [102,103,104,105,106,107,108,109,110]. Conversely, the induction of *NbSUS2*, *NbINV1*, *NbSWT12* and a cytosolic pyruvate kinase suggests mobilization of soluble carbon and maintenance of glycolytic energy supply [111,112,113,114,115,116]. In addition, the downregulation of classical protective genes, including *NbGSTX1/4* and LEA/dehydrin-related transcripts, indicates that *FaAP2-11* overexpression does not simply activate a conventional antioxidant or osmoprotective response [117,118]. Rather, the combined repression of photosynthetic machinery and selected stress-protective genes supports a restrictive, energy-saving metabolic state.

**Cell wall and surface remodeling**. Cluster D was associated with cell wall composition, wall remodeling and cell-surface-related processes. This module included genes related to hemicellulose remodeling, pectin modification, cellulose-associated processes, structural wall integrity and cuticular wax or surface-lipid biosynthesis. The selective induction of genes such as *NbEXPA1*, *NbXTH32* and cell wall scaffold-associated proteins, together with the broader repression of genes involved in cuticle biosynthesis, primary wall expansion and polysaccharide remodeling [119,120,121,122], indicates an altered transcriptional state associated with cell wall and cell-surface remodeling. This pattern is consistent with selective changes in wall properties rather than generalized activation of wall biosynthesis. In this context, the repression of several XTHs, cellulose synthase-like genes, pectin-related genes and cuticle-associated genes is consistent with reduced expansive growth and altered surface properties in *FaAP2-11*-overexpressing lines. However, these transcriptomic changes should be interpreted as associations with *FaAP2-11* activity rather than evidence of direct transcriptional regulation, as direct binding or promoter-level regulation of these genes by *FaAP2-11* was not assessed in the present study.

**Nutrient transport and selective detoxification.** Cluster E comprises genes related to osmoprotection, ion homeostasis, nutrient transport and detoxification. The expression pattern suggests selective reorganization of nutrient and ion transport rather than broad activation of classical dehydration-protection mechanisms. The induction of nitrate- and sulfate-transport-related genes, including *NbNTR31* and several *NbSULTR* copies, together with the repression of metal-associated transporters such as *NbHIP20*, points to a redirection of nutrient and ion fluxes [123,124,125]. Since nitrogen- and sulfur-containing compounds contribute to several classes of specialized stress-related metabolites, these changes may reflect redirection of nutrient fluxes towards selected protective metabolic pathways while reducing other energetically costly transport processes [126,127]. The repression of additional ion transporters, GST-related genes and LEA/dehydrin-related transcripts further supports the idea that *FaAP2-11* overexpression induces a selective, rather than generalized, osmoprotective response. Whereas Cluster C mainly reflects metabolic reprogramming, Cluster E primarily concerns nutrient allocation and transport processes.

**Specialized metabolism and defense-related responses**. Cluster F included genes associated with specialized metabolism and stress-associated defense responses. The expression profile suggested a reprogramming of phenylpropanoid, flavonoid/anthocyanin and triterpenoid-related metabolism. The induction of *NbMYBL4*, a negative regulator of flavonoid biosynthesis, together with the repression of *NbWRKY40* and *NbbHLH137*, suggests attenuation of the MBW-dependent flavonoid/anthocyanin regulatory branch [128,129,130,131]. At the same time, the induction of *NbUGT91A1* points to enhanced glycosylation of specialized metabolites, potentially facilitating their stabilization or vacuolar storage [132,133]. The induction of *NbBAMS* further suggests modulation of triterpenoid metabolism, which may contribute to surface-associated or defense-related compounds [134,135,136]. Finally, the repression of PP2C genes, which act as negative regulators of ABA signaling, together with the repression of ethylene-responsive transcription factors such as *NbERF25* and *NbERF106*, suggests a complex regulatory state in which ABA signaling may be facilitated while downstream ethylene-related stress transcriptional outputs are attenuated [12,25,137,138,139,140,141].

**Figure 12:**
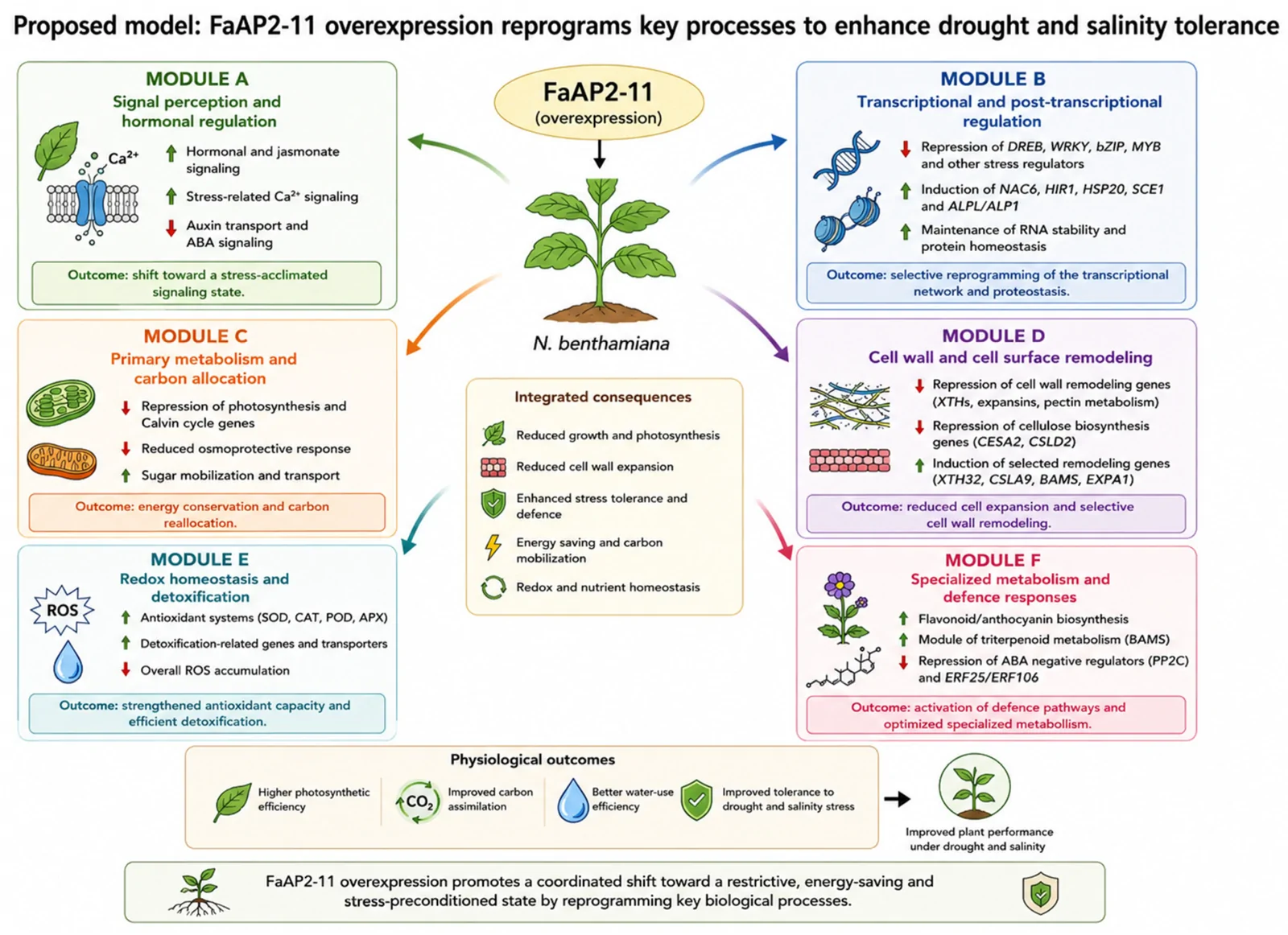
Proposed regulatory model underlying the role of *FaAP2-11* in drought and salinity stress tolerance. The model summarizes the main biological processes potentially regulated by *FaAP2-11* overexpression, including stress signaling, transcriptional and post-transcriptional regulation, primary metabolism and carbon allocation, cell wall remodeling, redox homeostasis and detoxification, and specialized metabolism and defense responses. Green arrows: Induction; Red arrows: Repression. The coordinated reprogramming of these processes is proposed to promote stress acclimation and ultimately enhance plant tolerance to drought and salinity stress. The figure was designed using artificial intelligence (AI) and subsequently adapted and edited by the authors.

Taken together, these six functional modules describe a coordinated transcriptional reprogramming induced by *FaAP2-11* overexpression (Figure 12). Rather than activating a classical stress regulon centered on DREB/LEA-associated responses, *FaAP2-11* appears to promote an alternative adaptive state characterized by hormonal rebalancing, selective attenuation of growth-associated processes, metabolic reorganization and optimization of physiological responses under osmotic stress. This integrated model is consistent with physiological analyses and supports the proposed involvement of *FaAP2-11* in the coordination of carbon metabolism, stress signaling and adaptive responses during drought and salinity.

Genes with ambiguous annotations or broad predicted functions were assigned to the most conservative category possible. Genes lacking sufficient functional information were classified as unknown or poorly annotated. This classification was not intended to replace statistical enrichment analyses, but rather to provide a supervised functional framework for interpreting the biological meaning of robust *FaAP2-11*-responsive genes. The complete gene-by-gene classification is provided in Supplementary Table S4. Collectively, the six functional modules support a coordinated transcriptional reprogramming in *FaAP2-11*-overexpressing plants characterized by hormonal rebalancing, attenuation of growth-associated processes, selective metabolic adjustment and optimization of stress-acclimation mechanisms. Rather than activating the classical DREB/LEA stress regulon, *FaAP2-11* appears to establish an alternative adaptive state in which carbon metabolism, signaling pathways and cellular protection are coordinately reconfigured to improve physiological performance under osmotic stress.

Importantly, the interpretation of these results should take into account the heterologous nature of the experimental system. Although *Nicotiana benthamiana* provides a valuable platform for obtaining functional evidence on the activity of *FaAP2-11*, the physiological and transcriptomic responses identified in this species cannot be assumed to fully recapitulate those occurring in *Fragaria × ananassa*. The octoploid genome and complex genomic background of cultivated strawberry may result in functional redundancy or subfunctionalization among homoeologous genes, while differences in transcriptional regulation, protein–protein interactions and downstream regulatory networks may influence the responses associated with *FaAP2-11* activity. Therefore, the DEGs, functional modules and physiological responses identified here should be considered as supportive evidence of a potential role of *FaAP2-11* in stress acclimation and cell wall remodeling, rather than as a definitive representation of its regulatory network in strawberry. Further functional studies directly in *F. × ananassa* will be required to validate these regulatory relationships and determine the extent to which the responses observed in *N. benthamiana* are conserved in strawberry.

## 3. Materials and Methods

### 3.1. Plant Material

Strawberry plants (*Fragaria × ananassa* Duch. cv. Chandler), an octoploid cultivar, were cultivated in a growth chamber maintained at 25 °C, 75% relative humidity (RH), and a 16 h light/8 h dark photoperiod. Relative humidity was modified only during stress experiments, according to the requirements of each treatment.

For all subsequent analyses, fully expanded leaves and roots were harvested, immediately frozen in liquid nitrogen, and stored at −80 °C until use.

### 3.2. Identification of AP2/ERF Family Members

Genome assembly and annotation data for *Fragaria vesca v4.0.a2* were retrieved from the Genome Database for *Rosaceae* (GDR, https://www.rosaceae.org/), accessed on 5 May 2026 [142]. Protein sequence alignments were performed using Clustal Omega (https://www.ebi.ac.uk/jdispatcher) [143], and conserved domains and functional sites were predicted using InterPro accessed on 5 May 2026 [144]. Domain predictions were subsequently validated using the NCBI Conserved Domain Database (CDD), followed by manual inspection of AP2 domain integrity to remove redundant sequences and identify candidate proteins containing at least one AP2 domain. Genome sequence and annotation data for *Arabidopsis thaliana* were obtained from Phytozome14 database (https://phytozome-next.jgi.doe.gov/, accessed on 6 May 2026) [144] and processed following the same procedure.

Phylogenetic analysis was performed using MEGA12 v12.0 [145]. The conserved AP2 domain sequences from the 58 AP2/ERF proteins identified in *Fragaria vesca* and 140 AP2/ERF proteins from *Arabidopsis thaliana* were aligned and used for phylogenetic reconstruction. A Neighbor-Joining (NJ) tree was generated with 1000 bootstrap replicates. The resulting phylogenetic tree was visualized and annotated using iTOL v7 [146]. Subcellular localization was predicted using LOCALIZER v1.0.4 accessed, on 10 May 2026 [147].

### 3.3. Chromosomal Localization and Gene Collinearity Analysis

Genome sequence and annotation files for *Fragaria vesca* v4.0.a2 and *Fragaria × ananassa* cv. Royal Royce v1.0 were retrieved from the Genome Database for *Rosaceae* (GDR) accessed on 5 May 2026 [142]. Synteny and collinearity analyses between *F. vesca* and *F. × ananassa* were performed using MCScanX with default parameters and an E-value threshold of ≤1 × 10^−10^ [148]. Collinearity relationships among the selected AP2 candidate genes were identified and visualized using TBtools-II v2.154 program [149].

### 3.4. Study of Cis-Acting Elements

The 2000 bp genomic regions upstream of the translation start site (ATG) of the selected FaAP2 genes were retrieved from the *F. vesca* v4.0.a2 genome through Phytozome14 database (https://phytozome-next.jgi.doe.gov/, accessed on 5 May 2026) [144]. These promoter sequences were analyzed using PlantCARE [150] (http://bioinformatics.psb.ugent.be/webtools/plantcare/html/, accessed on 15 May 2026), to identify putative *cis*-acting regulatory elements. The resulting data were processed and visualized using TBtools-II v2.154 program [149].

### 3.5. Stress Treatment in Strawberry Plants

Experiments were conducted using *Fragaria × ananassa* Duch. cv. Chandler plants to evaluate the effects of drought-like osmotic stress and salt stress. Prior to treatment, plants were transferred to sterile pots containing geotextile (GEOTEXAN, S.A.U, Spain) as an inert support substrate and supplied with 0.5× Murashige and Skoog (MS) medium (Duchefa Biochemie B.V., Haarlem, The Netherlands). Plants were acclimated to these conditions for 24 h at 70% relative humidity.

Drought-like osmotic stress was imposed by simultaneously reducing environmental RH to 30% and supplementing the 0.5× MS medium with 20% (*w*/*v*) polyethylene glycol 6000 (PEG 6000), thereby decreasing water availability to both aerial tissues and roots. Plants were maintained under these conditions for 5 h. Control plants were grown in 0.5× MS medium and maintained at 70% RH under the same environmental conditions.

For salt stress treatments, plants were acclimated under the same conditions described above. Salt stress was induced by supplementing the 0.5x MS medium with 200 mM NaCl for 72 h. Throughout the treatment period, plants were maintained at 70% RH in a controlled-environment chamber.

Relative humidity and temperature were continuously monitored during all experiments using a TRUTINA probe (ATMOS 14, METER Group Inc., Pullman, WA, USA). At the end of each treatment, leaves and roots were collected separately from control and stressed plants, immediately frozen in liquid nitrogen, and stored at −80 °C until further analysis.

### 3.6. Application of Pro-Oxidant Agents and Hormonal Regulators

To evaluate transcriptional responses to signaling molecules involved in stress tolerance, strawberry plants were treated with 5-aminolevulinic acid (ALA; 10 mg L^−1^), a known regulator of stress tolerance in strawberry [151], and sodium nitroprusside (SNP; 10 μM), used as a nitric oxide (NO) donor. Both compounds were prepared in 0.5x Murashige and Skoog (MS) medium and applied under controlled conditions. Leaf samples were collected 3 d after treatment.

Oxidative stress was induced by foliar application of hydrogen peroxide (H_2_O_2_; 100 mM) or paraquat (PQ; 1 mM). Solutions were prepared in sterile distilled water supplemented with 0.01% (*v*/*v*) Tween-20 to ensure uniform leaf coverage. Leaves treated with H_2_O_2_ were harvested 5 h after application, whereas PQ-treated leaves were collected after 6 and 24 h to evaluate both early and sustained stress responses. All experiments were conducted using three independent biological replicates.

Hormonal treatments were performed by foliar application of abscisic acid (ABA) and methyl jasmonate (MeJA), both at a final concentration of 0.1 mM, salicylic acid (SA) at 5 mM, and ethephon at 300 mg L^−1^. Treatment solutions were prepared in 5% (*v*/*v*) ethanol and supplemented with 0.01% (*v*/*v*) Tween-20 as a surfactant. Plants were sprayed using a manual atomizer to ensure uniform coverage of the leaf surface, and each plant received approximately 25 mL of the corresponding solution.

Control plants were treated with a solution containing 5% (*v*/*v*) ethanol and 0.01% (*v*/*v*) Tween-20 following the same procedure. Leaf samples were collected 6 h after treatment, immediately frozen in liquid nitrogen, and stored at −80 °C until further analysis.

### 3.7. RNA Extraction and Quality Assessment

The total RNA was isolated and purified from leaf and root samples of control strawberry plants and those subjected to water and salt stress. The Maxwell 16 Lev Plant RSC Instrument automated extraction system (manufactured by Promega Corporation, Madison, WI, USA) and the Maxwell RSC RNA FFPE kit (AS1440, Promega, Madison, WI, USA) were utilized, employing CTAB buffer (Promega, Madison, WI, USA). The process was carried out in accordance with the manufacturer’s instructions. In the final step, the RNA was eluted in 50 μL of RNase-free water and quantified with a Quantus^TM^ fluorometer using the Quantifluor^TM^ RNA kit (Promega, Madison, WI, USA). Quantification was performed in strict accordance with the manufacturer’s instructions, employing 1–20 µL of sample diluted in 200 µL of Quantifluor^TM^ RNA DYE working solution. The quality and integrity of the RNA were then subjected to rigorous assessed using an Agilent 2100 bioanalyzer (Agilent Technologies, Santa Clara, CA, USA).

### 3.8. Transcriptome Data Processing and Expression Profiling

For transcriptomic analysis, total RNA was extracted from leaves and roots of both control and stress-treated plants. RNA quantity and quality were assessed using a NanoDrop spectrophotometer (Bio-Rad, Hercules, CA, USA), agarose gel electrophoresis, and an Agilent 2100 Bioanalyzer (Agilent Technologies, Santa Clara, CA, USA). All samples exhibited an RNA Integrity Number (RIN) greater than 7. Three independent biological replicates were included for each experimental condition.

RNA-seq libraries with insert sizes ranging from 150 to 200 bp were constructed and sequenced on an Illumina HiSeq 2000 platform at Novogene Technology Co., Ltd. (Cambridge, UK). More than 80 million reads were generated per sample. Clean reads were aligned to the *Fragaria vesca* v2.0.a4 reference genome using TopHat2. Gene expression levels were quantified as fragments per kilobase of transcript per million mapped reads (FPKM).

Differentially expressed genes (DEGs) were identified by comparing normalized read counts between control and stress-treated samples. Statistical significance was assessed using a negative binomial model, and genes with an adjusted *p*-value < 0.05 were considered significantly differentially expressed. Additional filtering criteria included FPKM ≥ 5 and an absolute fold change ≥2.

### 3.9. Selection of AP2/ERF Transcription Factor Genes and Expression Profiling

AP2/ERF transcription factor-encoding genes were selected based on previous RNA-seq studies conducted by our research group in leaf and root tissues of strawberry plants (*Fragaria × ananassa* cv. Chandler) subjected to drought and salt stress, according to their differential expression profiles (Supplementary Table S1).

RNA-seq expression data were validated by quantitative reverse transcription PCR (RT-qPCR) using a CFX Real-Time PCR Detection System (Bio-Rad, Hercules, CA, USA). Gene-specific primers for the selected *FaAP2* genes were designed using the Primer-BLAST v2.5.0 tool available through the NCBI database (https://ncbi.nlm.nih.gov/tools/primer-blast/index.cgi, accessed on 6 August 2026) (Supplementary Table S5).

For each tissue type and experimental condition, at least three independent biological replicates were used for RNA extraction and purification. First-strand cDNA was synthesized from 2 μg of total RNA using the iScript™ Reliance Select cDNA Synthesis Kit (Bio-Rad, Hercules, CA, USA), following the manufacturer’s instructions. Quantitative PCR reactions were performed using SsoAdvanced™ Universal SYBR^®^ Green Supermix (Bio-Rad, Hercules, CA, USA) with 25 ng of cDNA per reaction.

All reactions were performed in triplicate, and amplification specificity was confirmed by melting curve analysis. Relative gene expression levels were calculated using the 2^−ΔΔCt^ method [152]. The constitutively expressed *FaGAPDH2* or *FaEF1α* gene was used as the internal reference for normalization, depending on the experimental condition.

### 3.10. Heterologous Transformation of the FaAP2-11 Gene in Nicotiana benthamiana

The complete coding sequence (CDS) of *FaAP2-11* was obtained from Phytozome [144]. The sequence was synthesized by GenScript (Piscataway, NJ, USA) and cloned into the pK7WG2 overexpression vector (https://vectorvault.vib.be/), which contains the CaMV35S promoter, generating the pK7WG2::*FaAP2-11* construct (*FaAP2-11-OX*).

Stable transformation of *Nicotiana benthamiana* was performed using *Agrobacterium tumefaciens* strain GV3101 harboring either the *FaAP2-11-OX* construct or the empty pK7WG2 vector, which served as the transgenic control [153]. Stable transformation of *Nicotiana benthamiana*. Agrobacterium cultures were grown in LB medium supplemented with the appropriate antibiotics and 20 μM acetosyringone at 28 °C for 24–48 h. Cells were harvested by centrifugation and resuspended in MMA infiltration medium (4.3 g L^−1^ MS salts, 20 g L^−1^ sucrose, and 10 mM MES, pH 5.7–5.8) to an OD600 of 0.8.

Leaf explants (1 × 1 cm) excised from 3–5-week-old *N. benthamiana* plants were immersed in the bacterial suspension for 30 min. Following inoculation, explants were blotted dry and incubated on moist filter paper for 48 h in darkness for co-cultivation. Explants were then washed with sterile water containing kanamycin (100 mg L^−1^) and cefotaxime (250 mg L^−1^).

After drying, explants were transferred to MS-II regeneration medium containing 4.3 g L^−1^ MS salts, 1 mg L^−1^ 6-benzylaminopurine (BAP), and 0.1 mg L^−1^ α-naphthaleneacetic acid (NAA), supplemented with kanamycin and cefotaxime. Cultures were maintained in a growth chamber at 25 °C under a 16 h light/8 h dark photoperiod with a light intensity of 105–125 μmol m^−2^ s^−1^ until shoot regeneration occurred.

Regenerated shoots were transferred to MS-III rooting medium containing 4.3 g L^−1^ MS salts, 100 mg L^−1^ kanamycin, and 200 mg L^−1^ cefotaxime. Plantlets were maintained in this medium until adequate root and shoot development was achieved. Subsequently, plants were acclimated and transferred to soil. RT-qPCR analysis was performed to identify transgenic lines exhibiting elevated *FaAP2-11* expression.

### 3.11. Determination of Physiological Parameters in Nicotiana benthamiana FaAP2-11-OX Transgenic Plants

To assess the physiological response of *Nicotiana benthamiana FaAP2-11-OX* plants, three independent transgenic lines 8, 14 and 17, representing distinct transformation events, were evaluated alongside control plants carrying the empty vector (two independent lines). Six biological replicates were measured per line. Gas exchange measurements were performed on the youngest fully expanded leaves using a LI-6400/XT Portable Photosynthesis System (LI-COR Biosciences, Lincoln, NE, USA) equipped with the 6400-01 CO_2_ injector and the leaf chamber fluorometer 6400-40 LCF. Parameters were established in 400 µmol mol^−1^ CO_2_, flow of 350 µmol s^−1^ and PAR in 1000 µmol m^−2^ s^−1^.

Three technical repetitions were measured from each biological replicate, allowing a stabilization time of 75 s for each measurement. Following this, net assimilation (A, μmol CO_2_ m^−2^ s^−1^), stomatal conductance (Gs, molH_2_O m^−2^ s^−1^) relative CO_2_ concentration inside the leaf (Ci, µmol CO_2_ mol CO_2_^−1^), maximum photosynthetic efficiency of photosystem II (Qy, dimensionless), vapour pressure deficit in leaves (VpdL, kPa) and water use efficiency (WUE, μmol CO_2_ mol^−1^ H_2_O) were calculated and analyzed. For the statistical analysis, normal distribution of the data was checked using the Anderson–Darling test. Then, variables which did not fit normal distribution were log-transformed for statistical analysis and variance was assessed using ANOVA test. Differences between means were checked by means of the Tukey post-hoc test for the least significance difference (LSD) threshold. Significance level was set at *p* = 0.05 for all the statistical procedures. The analysis of all the physiological variables was carried out in the R environment (R Core Team, 2022) using the RStudio user interface (RStudio Team, 2026 01.1 +403).

### 3.12. Transcriptome Analysis of FaAP2-11 Overexpression Lines

To investigate the transcriptional changes associated with *FaAP2-11* overexpression, RNA-seq analysis was performed using three independent *Nicotiana benthamiana* transgenic lines exhibiting the highest levels of *FaAP2-11* expression (L8, L14, and L17). Three independent empty-vector lines (EV1, EV2, and EV3) were used as controls. Two biological replicates were analyzed for each line, resulting in a total of six biological replicates for both the overexpression and control groups. Young leaves at the same developmental stage were collected from all plants for RNA extraction.

RNA quantity and quality were assessed using a NanoDrop spectrophotometer, agarose gel electrophoresis, and an Agilent 2100 Bioanalyzer. All samples exhibited an RNA Integrity Number (RIN) greater than 7. RNA-seq libraries with insert sizes ranging from 150 to 200 bp were constructed and sequenced on an Illumina HiSeq 2000 platform at Novogene Europe (Cambridge, UK) generating more than 80 million reads per sample.

Clean reads were mapped to the *Nicotiana benthamiana v2.6.1* reference genome, and gene expression levels were quantified as fragments per kilobase of transcript per million mapped reads (FPKM). Differential gene expression analysis was performed using DESeq2 (v1.20.0) and *p*-values were adjusted using the Benjamini–Hochberg method. Differentially expressed genes (DEGs) between *FaAP2-11*-overexpressing lines and empty-vector controls were identified using an adjusted *p*-value threshold of < 0.05 and a fold-change cutoff of ≥ 2. Subsequent analyses were performed using the resulting DEG dataset.

For functional interpretation, a subset of robustly regulated genes was selected using additional filtering criteria: padj ≤ 0.05, FPKM ≥ 5 and a fold-change cutoff of ≥ 3 for upregulated genes or ≤1/3 for downregulated genes. Upregulated and downregulated genes were analyzed separately. This filtering strategy identified 314 differentially expressed genes, of which 67 were upregulated and 247 were downregulated. RNA-seq data have been deposited in the public GEO database GSE342796.

To facilitate the biological interpretation of the RNA-seq dataset, filtered DEGs were manually organized into putative functional modules based on available *Nicotiana benthamiana* gene annotation, predicted biological function, gene family information, conserved functional descriptions and, when available, the reported biological roles of homologous plant genes. This curated classification was used as a biologically guided framework to group genes into major biological processes potentially affected by *FaAP2-11* overexpression.

The resulting DEG dataset was organized into six putative functional modules and 24 functional subgroups, as detailed in Supplementary Table S4.1 and S4.2. Each gene was assigned to a single primary functional module according to its most likely predominant biological function. Genes with ambiguous annotations or broad predicted functions were assigned to the most conservative category possible, whereas genes lacking sufficient functional information were classified as unknown or poorly annotated.

This classification was not intended to replace formal functional enrichment analyses, such as GO or KEGG enrichment, but rather to provide a supervised interpretative framework for downstream biological interpretation and candidate gene selection.

## 4. Conclusions

This study provides a comprehensive characterization of drought- and salinity-responsive AP2/ERF transcription factors in strawberry and identifies *FaAP2-11* as an important regulator involved in abiotic stress acclimation. Phylogenetic analysis revealed that most stress-responsive genes belong to the ERF family, whereas expression analyses uncovered partially redundant and tissue-specific regulatory clusters integrating drought and salinity responses. In addition, promoter architecture and synteny analyses suggest that the functional and regulatory diversification of *AP2/ERF* genes has contributed to the adaptation of the octoploid strawberry genome to adverse environmental conditions.

Among the identified genes, *FaAP2-11* displayed a distinctive expression pattern, exhibiting strong responsiveness to hormone- and redox-related signals together with preferential accumulation in achenes and sepals, suggesting a functional link between developmental processes and stress acclimation. Heterologous overexpression of *FaAP2-11* in *Nicotiana benthamiana* resulted in improved photosynthetic performance and enhanced water-use efficiency under osmotic and salt stress conditions, demonstrating its positive contribution to physiological acclimation.

Furthermore, transcriptomic analyses suggest that *FaAP2-11* promotes a coordinated transcriptional shift towards a more restrictive and energy-saving physiological state, characterized by attenuation of photosynthesis, growth-associated pathways, canonical DREB/LEA-type stress responses, and primary cell wall expansion and cuticle-related processes, while selectively maintaining or activating hormone signaling, redox homeostasis, carbon allocation and specialized metabolism. Thus, *FaAP2-11* appears to function not as a simple activator of a conventional stress-response programme, but rather as a regulator of an alternative acclimation-like state that redirects cellular activity from growth-associated processes towards a more restrictive, energy-saving and stress-preconditioned physiological state.

Overall, our findings support an important regulatory role for *FaAP2-11* in coordinating the physiological and transcriptional responses associated with drought and salinity stress acclimation through the integration of hormonal, redox and metabolic signals.

This work provides new insights into AP2/ERF-mediated regulatory mechanisms in strawberry, identifies *FaAP2-11* as a promising candidate for future functional studies, and raises the possibility of its future application in crop-improvement strategies aimed at enhancing resilience to water deficit and salinity under climate change scenarios, pending direct functional validation in *Fragaria × ananassa*.

## Figures and Tables

**Table 1:** Functional classification of the 314 high-confidence differentially expressed genes (DEGs) identified in leaves of *FaAP2-11*-overexpressing *Nicotiana benthamiana* plants following stringent filtering (adjusted *p* ≤ 0.05, FPKM ≥ 5 and |fold change| ≥ 3). DEGs were manually grouped into six putative functional modules based on gene annotation, predicted biological function and available literature. Each module summarizes the number of totals, upregulated and downregulated genes, together with the predominant transcriptional trends, their putative biological interpretation, and the proposed relationship between *FaAP2-11* overexpression and the affected biological processes.

Putative Functional Module	Total Genes	UP	DOWN	Biological Interpretation	Relationship Among Groups
Cluster A. Environmental perception and signal transduction	59	13	46	Repression of Ca^2+^ and cell wall sensors/transducers predominates, together with selective hormonal changes. This pattern suggests a reprogrammed basal signaling state rather than a widespread activation of stress responses.	Links FaAP2/ERF11 overexpression to ABA, JA, auxin and GA signaling, as well as osmotic and mechanical sensing.
Cluster B. Transcriptional and post-transcriptional regulation	83	18	65	Transcriptional reprogramming was dominated by the repression of DREB, WRKY, bZIP, and MYB transcription factors and proteostasis-related components, with selective activation of NAC6, HIR1, HSP20, and SCE1	Supports the view that FaAP2/ERF11 does not activate a canonical DREB regulon, but instead selectively reprograms the transcriptional regulatory network.
Cluster C. Primary Metabolism Reprogramming, Redox Homeostasis and Detoxification	44	10	34	Strong repression of photosynthesis, the Calvin cycle and the classical osmoprotective response, while maintaining sugar mobilization pathways and selected transport processes.	Supports an energy-conserving strategy involving carbon reallocation in FaAP2/ERF11-overexpressing lines.
Cluster D. Cell Wall Composition and Remodeling	68	8	60	Extensive repression of genes involved in cell wall remodeling (XTHs, CesA/CSL, pectin metabolism and expansins) and cuticular barrier formation, accompanied by the selective induction of XTH32, CSLA9, BAMS and EXPA1	Suggests growth restriction and reduced cell expansion, accompanied by selective remodeling of the cell wall and epidermal surface.
Cluster E. Radical Ions Protection and Detoxification	30	8	22	Mixed response with enhanced nitrogen and sulfur transport and selective activation of detoxification-related genes, but repression of antioxidant defense components and osmotic stress-responsive genes.	Suggests selective redox and ionic reprogramming rather than a broad activation of antioxidant defense mechanisms.
Cluster F. Specialized Metabolism Reprogramming and Genes involved in REDOX status	30	10	20	Reprogramming of phenylpropanoid and specialized metabolic pathways, together with repression of genes involved in redox homeostasis and cytochrome P450-mediated metabolism.	Supports coordinated reprogramming of secondary metabolism and redox homeostasis, potentially linked to cell wall remodeling.
TOTAL GENES	314	67	247		

**Table 2:** Integrative model of the transcriptional reprogramming associated with *FaAP2-11* overexpression in *Nicotiana benthamiana leaves*. The model summarizes the major biological processes inferred from the six putative functional modules identified among the high-confidence differentially expressed genes (DEGs). For each process, the principal supporting module, secondary connections with other modules, and the corresponding functional interpretation are indicated.

Integrative Model Process	Mainly Inferred from	Secondary Connections	Functional Interpretation
1. Hormonal reprogramming	Module A	—	Reflects changes in hormone metabolism and signaling, including auxin, gibberellin, ABA, jasmonate and brassinosteroid-related components.
2. Attenuation of stress sensors/transducers	Module A	Partial connection with Module D	Mainly reflects repression of Ca^2+^-dependent signaling, osmotic/mechanical sensing and plasma membrane-/cell wall-associated perception components.
3. Repression of the classical stress regulon	Module B	Supported by Module E through LEA/dehydrin-related genes	Mainly reflects repression of canonical stress-associated transcriptional regulators, especially DREB/CBF-related genes, together with reduced expression of classical protective genes.
4. Reduction in photosynthesis and growth	Module C	Partial support from Modules A and D	Reflects repression of photosynthetic machinery, primary metabolism and growth-associated processes, consistent with reduced investment in energetically costly functions.
5. Cell wall/cuticle remodeling	Module D	Connection with Module A through cell wall-associated sensing	Reflects selective changes in wall remodeling, wall integrity, polysaccharide-related processes and cuticle or surface-lipid biosynthesis.
6. Specialized metabolism and defence	Module F	Partial connections with Modules C, E and D	Reflects reprogramming of phenylpropanoid, flavonoid/anthocyanin, triterpenoid, glycosylation and defense-associated pathways, linked to carbon, nutrient and surface-related changes.

## Data Availability

The original contributions presented in this study are included in the article/Supplementary Material. Further inquiries can be directed at the corresponding author. RNA-seq data have been deposited in the public GEO database GSE342796.

## Supplementary Materials

The following supporting information can be downloaded at: https://www.mdpi.com/article/10.3390/ijms27188203/s1.

## Abbreviations

The following abbreviations are used in this manuscript:

TF	Transcription factor
ROS	Reactive oxygen species
RH	Relative humidity
PEG	Polyethylene glycol
PQ	Paraquat
SNP	sodium nitroprusside
ABA	abscisic acid
ALA	5-aminolevulinic acid
MeJA	methyl jasmonate
SA	salicylic acid
NO	nitric oxide
