# Supplementary material for: The FaAP2-11 Transcription Factor Putatively Regulates Cell Wall Remodeling and Stress-Acclimation Responses Under Drought and Salinity Stress
Source: Int J Mol Sci. 2026 Sep 15;27(18):8203. doi: 10.3390/ijms27188203 (PMC13607100; doi:10.3390/ijms27188203)
Supplement: Supplementary file 1 [file ijms-27-08203-s001.zip › Figures S1-S3.pdf]

The FaAP2-11 transcription factor putatively regulates cell wall remodeling and stress-acclimation responses under drought and salinity stress.

Supplementary Materials

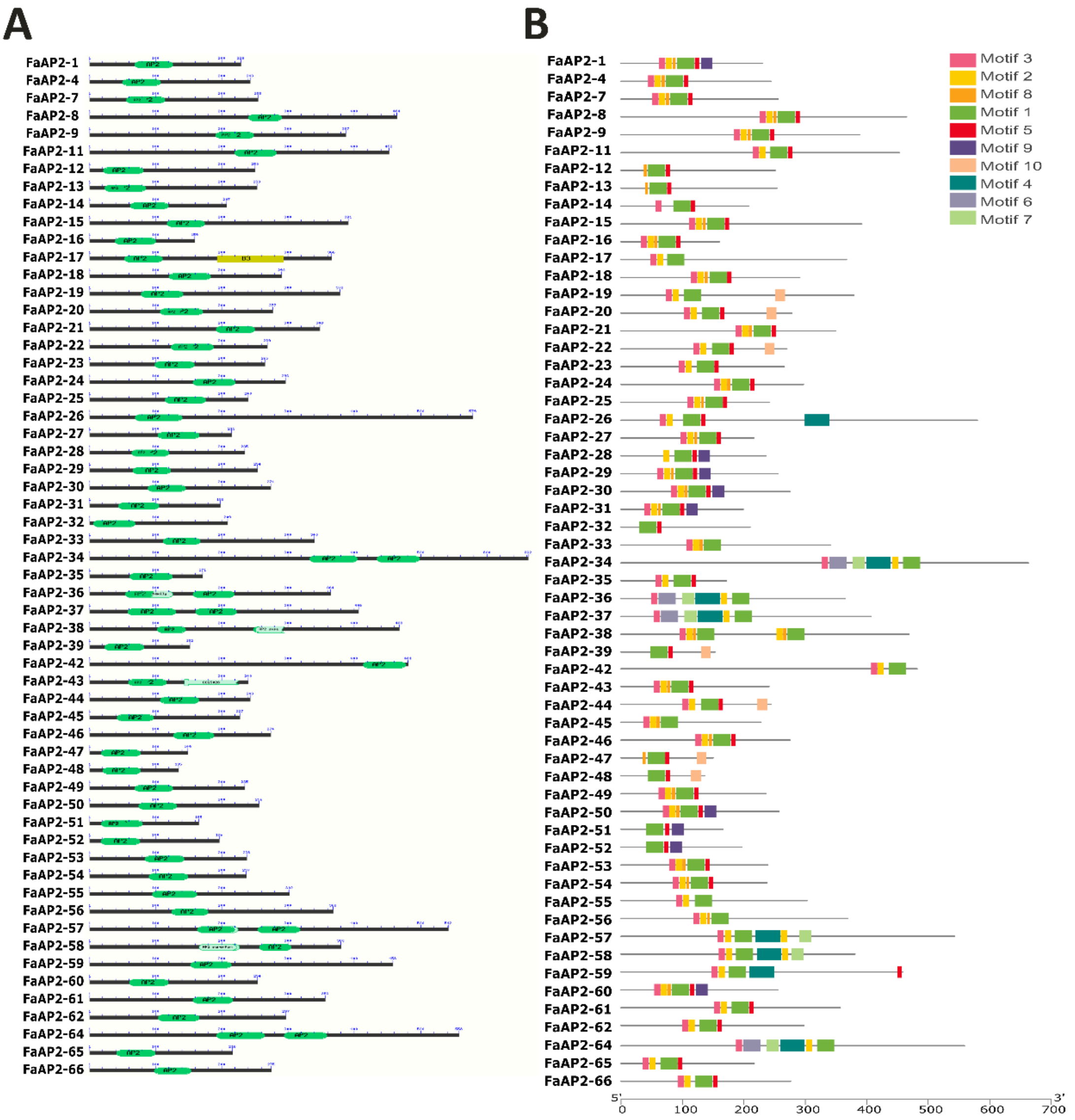

Figure S1. Conserved domain architecture and motif composition of FaAP2/ERF proteins. (A) Conserved domains identified using the NCBI Conserved Domain Database (CDD). Green boxes represent AP2 DNA-binding domains, whereas orange boxes indicate B3 DNA-binding domains. Members of the AP2 subfamily contain two AP2 domains, while RAV proteins possess both AP2 and B3 domains. Grey lines represent the full-length protein sequences. (B) Conserved motifs identified using MEME. Different colored boxes represent the ten conserved motifs (Motifs 1–10), as indicated in the legend, and grey lines correspond to the full-length protein sequences. Domain and motif positions are shown to scale.

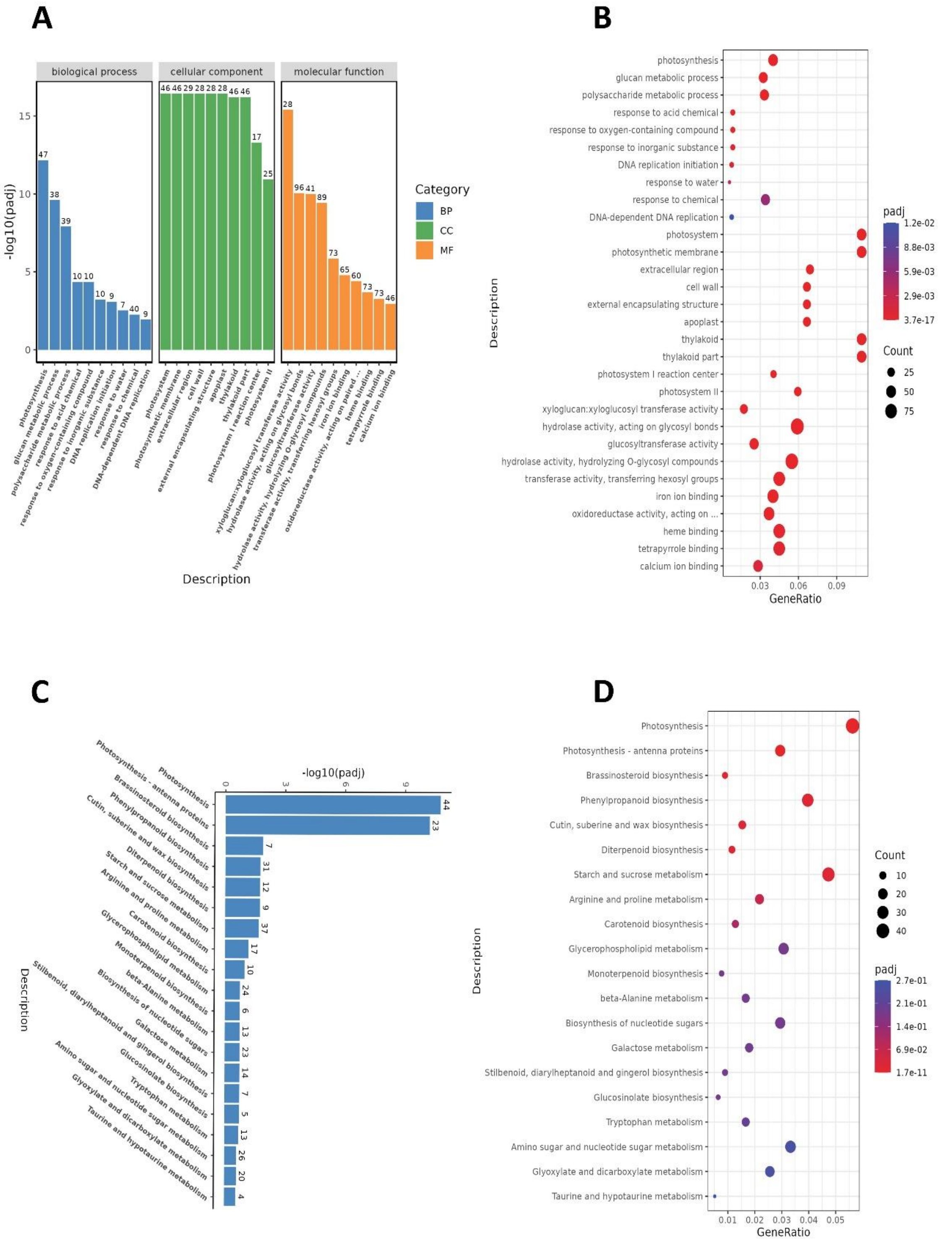

Figure S2. Functional enrichment analysis of differentially expressed genes (DEGs) identified in leaves of *FaAP2-11*-overexpressing *Nicotiana benthamiana* plants based on the initial differential expression analysis performed by Novogene (adjusted $p < 0.05$). (A) Gene Ontology (GO) enrichment bar plot showing the most significantly enriched terms within the Biological Process (BP), Cellular Component (CC), and Molecular Function (MF) categories. (B) GO enrichment dot plot displaying GeneRatio, number of DEGs (dot size), and adjusted P value (dot color) for the significantly enriched GO terms. (C) Kyoto Encyclopedia of Genes

and Genomes (KEGG) pathway enrichment bar plot showing the most significantly enriched pathways. (D) KEGG pathway enrichment dot plot illustrating GeneRatio, number of DEGs (dot size), and adjusted P value (dot color) for the enriched pathways.

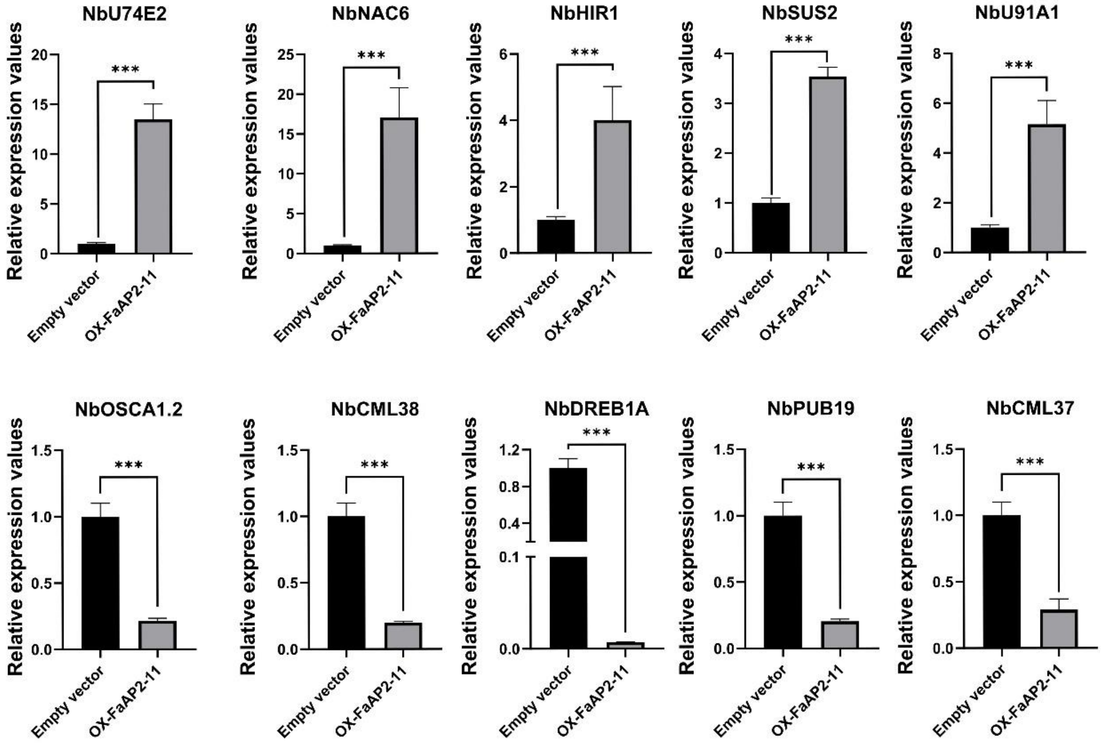

Figure S3. Validation of RNA-seq data by RT-qPCR in leaves of *Nicotiana benthamiana* overexpressing *FaAP2-11*. Relative transcript levels of ten representative differentially expressed genes were determined by RT-qPCR in leaves of empty vector and *FaAP2-11*-overexpressing (*FaAP2-11*-OX) plants. The selected genes include both upregulated (*NbUGT74E2, NbNAC6, NbHIR1, NbSUS2 and NbUGT91A1*) and downregulated (*NbOSCA1.2, NbCML38, NbDREB1A, NbPUB19 and NbCML37*) transcripts identified by RNA-seq. Gene expression was normalized against the reference gene *NbUBQ3* and is presented as the mean ± SD of three biological replicates. Asterisks indicate significant differences between empty vector and *FaAP2-11*-OX plants (Student's t-test; ***p < 0.005). The RT-qPCR expression patterns were consistent with those obtained by RNA-seq, confirming the reliability of the transcriptomic dataset.
