# Supplementary material for: The FaAP2-11 Transcription Factor Putatively Regulates Cell Wall Remodeling and Stress-Acclimation Responses Under Drought and Salinity Stress
Source: Int J Mol Sci. 2026 Sep 15;27(18):8203. doi: 10.3390/ijms27188203 (PMC13607100; doi:10.3390/ijms27188203)
Supplement: Supplementary file 1 [file ijms-27-08203-s001.zip › Tables S1-3 y S5.pdf]

The FaAP2-11 transcription factor putatively regulates cell wall remodeling and stress-acclimation responses under drought and salinity stress.

Supplementary Materials

Table S1. Differentially expressed *FaAP2/ERF* transcription factor genes identified in leaf and root tissues under drought and salinity stress**.** Values represent gene expression fold changes in upregulated (UP) and downregulated (DOWN) genes. The closest homolog identified by sequence similarity is indicated in the Description column. (UP, upregulated; DOWN, downregulated; N.D., not detected (no differentially expressed gene identified under the corresponding condition).

	DROUGHT				SALINITY				
	UP		DOWN		UP		DOWN		
**Gene name**	**LEAF**	**ROOT**	**LEAF**	**ROOT**	**LEAF**	**ROOT**	**LEAF**	**ROOT**	**DESCRITPION**
FaAP2-1	156,3	N.D	N.D	N.D	47,33	31,29	N.D	N.D	|Q9FJ93|DRE1D_ARATH Dehydration-responsive element-binding protein 1D OS=Arabidopsis thaliana ON. D=3702 GN=DREB1D PE=2 SV=1 && PF00847:AP2 domain
FaAP2-4	9,52	N.D	N.D	N.D	62,50	3,03	N.D	N.D	|Q9LY29|EF115_ARATH Ethylene-responsive transcription factor ERF115 OS=Arabidopsis thaliana ON. D=3702 GN=ERF115 PE=1 SV=1 && PF00847:AP2 domain
FaAP2-7	5,24	N.D	N.D	N.D	16,00	3,23	N.D	N.D	|Q9FH54|EF114_ARATH Ethylene-responsive transcription factor ERF114 OS=Arabidopsis thaliana ON. D=3702 GN=ERF114 PE=1 SV=1 && PF00847:AP2 domain
FaAP2-8	309,5	3,61	N.D	N.D	399,49	15,83	N.D	N.D	|Q70II3|EF110_ARATH Ethylene-responsive transcription factor ERF110 OS=Arabidopsis thaliana ON. D=3702 GN=ERF110 PE=2 SV=2 && PF00847:AP2 domain
FaAP2-9	20,76	3,57	N.D	N.D	361,56	14,18	N.D	N.D	|Q9FGF8|ABR1_ARATH Ethylene-responsive transcription factor ABR1 OS=Arabidopsis thaliana ON. D=3702 GN=ABR1 PE=2 SV=1 && PF00847:AP2 domain

FaAP2-11	205	N.D	N.D	14,57	92,16	N.D	N.D	N.D	ǀQ9SKT1ǀERF53_ARATH Ethylene-responsive transcription factor ERF053 OS=Arabidopsis thaliana ON. D=3702 GN=ERF053 PE=1 SV=1 && PF00847:AP2 domain
FaAP2-12	7,70	N.D	N.D	N.D	N.D	N.D	N.D	N.D	ǀO80340ǀERF78_ARATH Ethylene-responsive transcription factor 4 OS=Arabidopsis thaliana ON. D=3702 GN=ERF4 PE=1 SV=1 && PF00847:AP2 domain
FaAP2-13	7,82	N.D	N.D	N.D	5,03	2,07	N.D	N.D	ǀQ9LW49ǀERF4_NICSY Ethylene-responsive transcription factor 4 OS=Nicotiana sylvestris ON. D=4096 GN=ERF4 PE=2 SV=1 && PF00847:AP2 domain
FaAP2-14	519,4	N.D	N.D	N.D	3,58	N.D	N.D	N.D	ǀP93835ǀDRE1B_ARATH Dehydration-responsive element-binding protein 1B OS=Arabidopsis thaliana ON. D=3702 GN=DREB1B PE=1 SV=2 && PF00847:AP2 domain
FaAP2-15	3,99	N.D	N.D	N.D	3,37	2,25	N.D	N.D	ǀQ9LUM4ǀRAP22_ARATH Ethylene-responsive transcription factor RAP2-2 OS=Arabidopsis thaliana ON. D=3702 GN=RAP2-2 PE=1 SV=2 && PF00847:AP2 domain
FaAP2-16	2,88	2,07	N.D	N.D	N.D	N.D	N.D	N.D	ǀQ9SNE1ǀERF11_ARATH Ethylene-responsive transcription factor ERF011 OS=Arabidopsis thaliana ON. D=3702 GN=ERF011 PE=2 SV=1 && PF00847:AP2 domain
FaAP2-17	3,02	2,98	N.D	N.D	N.D	N.D	N.D	N.D	ǀP82280ǀRAV2_ARATH AP2/ERF and B3 domain-containing transcription repressor RAV2 OS=Arabidopsis thaliana ON. D=3702 GN=RAV2 PE=1 SV=1 && PF00847:AP2 domainǀPF02362:B3 DNA binding domain
FaAP2-18	2,78	N.D	N.D	N.D	N.D	6,18	N.D	N.D	ǀQ40478ǀERF5_TOBAC Ethylene-responsive transcription factor 5 OS=Nicotiana tabacum ON. D=4097 GN=ERF5 PE=2 SV=1 && PF00847:AP2 domain
FaAP2-19	23,63	N.D	N.D	N.D	38,33	25,67	N.D	N.D	ǀO82132ǀDRE2A_ARATH Dehydration-responsive element-binding protein 2A OS=Arabidopsis thaliana ON. D=3702 GN=DREB2A PE=1 SV=1 && PF00847:AP2 domain
FaAP2-20	69,19	N.D	N.D	N.D	26,94	7,15	N.D	N.D	ǀA0A3Q7I5Y9ǀERFC3_SOLLC Ethylene-response factor C3 OS=Solanum lycopersicum ON. D=4081 GN=ERF.C.3 PE=2 SV=1 && PF00847:AP2 domain
FaAP2-21	3,36	2,80	N.D	N.D	2,33	2,52	N.D	N.D	ǀO80341ǀEF102_ARATH Ethylene-responsive transcription factor 5 OS=Arabidopsis thaliana ON. D=3702 GN=ERF5 PE=2 SV=1 && PF00847:AP2 domain

FaAP2-22	146,8	N.D	N.D	N.D	388,07	8,30	N.D	N.D	ǀA0A3Q7I5Y9ǀERFC3_SOLLC Ethylene-response factor C3 OS=Solanum lycopersicum ON.D=4081 GN=ERF.C.3 PE=2 SV=1 && PF00847:AP2 domain
FaAP2-23	5,46	N.D	N.D	2,74	N.D	10,67	N.D	N.D	ǀO04682ǀPTI6_SOLLC Pathogenesis-related genes transcriptional activator PTI6 OS=Solanum lycopersicum ON. D=4081 GN=PTI6 PE=2 SV=1 && PF00847:AP2 domain
FaAP2-24	11,69	2,70	N.D	N.D	4,70	5,50	N.D	N.D	ǀO80337ǀEF100_ARATH Ethylene-responsive transcription factor 1A OS=Arabidopsis thaliana ON. D=3702 GN=ERF1A PE=1 SV=2 && PF00847:AP2 domain
FaAP2-25	N.D	5,26	N.D	N.D	138,03	18,83	N.D	N.D	ǀQ9SZ06ǀEF109_ARATH Ethylene-responsive transcription factor ERF109 OS=Arabidopsis thaliana ON. D=3702 GN=ERF109 PE=1 SV=1 && PF00847:AP2 domain
FaAP2-26	N.D	2,28	N.D	N.D	145,57	41,57	N.D	N.D	ǀO80917ǀDRE2E_ARATH Dehydration-responsive element-binding protein 2E OS=Arabidopsis thaliana ON. D=3702 GN=DREB2E PE=2 SV=1 && PF00847:AP2 domain
FaAP2-27	N.D	2,37	N.D	N.D	N.D	2,07	7,87	N.D	ǀQ9FKG2ǀEF107_ARATH Ethylene-responsive transcription factor ERF107 OS=Arabidopsis thaliana ON. D=3702 GN=ERF107 PE=2 SV=1 && PF00847:AP2 domain
FaAP2-28	N.D	2,31	N.D	N.D	16,05	5,27	N.D	N.D	ǀQ9CAN9ǀERF26_ARATH Ethylene-responsive transcription factor ERF026 OS=Arabidopsis thaliana ON. D=3702 GN=ERF026 PE=2 SV=1 && PF00847:AP2 domain
FaAP2-29	N.D	N.D	2,21	N.D	N.D	N.D	68,99	2,20	ǀQ9LYD3ǀDREB3_ARATH Dehydration-responsive element-binding protein 3 OS=Arabidopsis thaliana ON. D=3702 GN=DREB3 PE=2 SV=1 && PF00847:AP2 domain
FaAP2-30	N.D	N.D	4,46	N.D	N.D	N.D	13,55	N.D	ǀQ8LBQ7ǀERF34_ARATH Ethylene-responsive transcription factor ERF034 OS=Arabidopsis thaliana ON. D=3702 GN=ERF034 PE=2 SV=2 && PF00847:AP2 domain
FaAP2-31	N.D	N.D	6,35	N.D	N.D	N.D	17,78	N.D	ǀQ1ECI2ǀERF23_ARATH Ethylene-responsive transcription factor ERF023 OS=Arabidopsis thaliana ON. D=3702 GN=ERF023 PE=2 SV=1 && PF00847:AP2 domain
FaAP2-32	N.D	N.D	N.D	2,81	N.D	N.D	N.D	3,44	ǀQ94AW5ǀERF03_ARATH Ethylene-responsive transcription factor ERF003 OS=Arabidopsis thaliana ON. D=3702 GN=ERF003 PE=1 SV=1 && PF00847:AP2 domain

FaAP2-33	N.D	N.D	N.D	2,56	N.D	N.D	N.D	N.D	ǀQ9SUQ2ǀCRF2_ARATH Ethylene-responsive transcription factor CRF2 OS=Arabidopsis thaliana ON. D=3702 GN=CRF2 PE=1 SV=1 && PF00847:AP2 domain
FaAP2-34	N.D	N.D	N.D	4,01	N.D	N.D	N.D	12,14	ǀQ38914ǀANT_ARATH AP2-like ethylene-responsive transcription factor ANT OS=Arabidopsis thaliana ON. D=3702 GN=ANT PE=1 SV=2 && PF00847:AP2 domain
FaAP2-35	19,35	N.D	N.D	N.D	427,58	25,35	N.D	N.D	ǀO04681ǀPTI5_SOLLC Pathogenesis-related genes transcriptional activator PTI5 OS=Solanum lycopersicum ON. D=4081 GN=PTI5 PE=2 SV=1 && PF00847:AP2 domain
FaAP2-36	N.D	N.D	N.D	N.D	18,48	N.D	N.D	N.D	ǀQ94AN4ǀAP2L1_ARATH AP2-like ethylene-responsive transcription factor At1g16060 OS=Arabidopsis thaliana ON. D=3702 GN=At1g16060 PE=2 SV=1 && PF00847:AP2 domain
FaAP2-37	N.D	N.D	N.D	N.D	68,36	N.D	N.D	N.D	ǀQ6N.D5Y6ǀWRI1_ARATH Ethylene-responsive transcription factor WRI1 OS=Arabidopsis thaliana ON. D=3702 GN=WRI1 PE=1 SV=1 && PF00847:AP2 domain
FaAP2-38	N.D	N.D	N.D	N.D	2,35	N.D	N.D	N.D	ǀQ9CA27ǀEF118_ARATH Ethylene-responsive transcription factor ERF118 OS=Arabidopsis thaliana ON. D=3702 GN=ERF118 PE=1 SV=1 && PF00847:AP2 domain
FaAP2-39	N.D	N.D	N.D	N.D	89,54	N.D	N.D	N.D	ǀQ9LTC5ǀERF98_ARATH Ethylene-responsive transcription factor ERF098 OS=Arabidopsis thaliana ON. D=3702 GN=ERF098 PE=1 SV=1 && PF00847:AP2 domain
FaAP2-42	N.D	N.D	N.D	N.D	2,58	2,30	N.D	N.D	ǀQ9SUE3ǀCRF4_ARATH Ethylene-responsive transcription factor CRF4 OS=Arabidopsis thaliana ON. D=3702 GN=CRF4 PE=1 SV=2 && PF03478: Protein of unknown function (DUF295)
FaAP2-43	N.D	N.D	N.D	N.D	265,95	2,82	N.D	N.D	ǀQ9FH54ǀEF114_ARATH Ethylene-responsive transcription factor ERF114 OS=Arabidopsis thaliana ON. D=3702 GN=ERF114 PE=1 SV=1 && PF00847:AP2 domain
FaAP2-44	N.D	N.D	N.D	N.D	121,28	N.D	N.D	N.D	ǀQ8LDC8ǀERF92_ARATH Ethylene-responsive transcription factor 1B OS=Arabidopsis thaliana ON. D=3702 GN=ERF1B PE=1 SV=2 && PF00847:AP2 domain

FaAP2-45	N.D	N.D	N.D	N.D	2,52	N.D	N.D	N.D	|Q9SN.DS8|ERF3_TOBAC Ethylene-responsive transcription factor 3 OS=Nicotiana tabacum ON. D=4097 GN=ERF3 PE=2 SV=1 && PF00847:AP2 domain
FaAP2-46	N.D	N.D	N.D	N.D	N.D	45,16	N.D	N.D	|Q9SZ06|EF109_ARATH Ethylene-responsive transcription factor ERF109 OS=Arabidopsis thaliana ON. D=3702 GN=ERF109 PE=1 SV=1 && PF00847:AP2 domain
FaAP2-47	N.D	N.D	N.D	N.D	N.D	63,15	N.D	N.D	|Q9LTC5|ERF98_ARATH Ethylene-responsive transcription factor ERF098 OS=Arabidopsis thaliana ON. D=3702 GN=ERF098 PE=1 SV=1 && PF00847:AP2 domain
FaAP2-48	N.D	N.D	N.D	N.D	N.D	31,68	N.D	N.D	|Q9LTC6|ERF95_ARATH Ethylene-responsive transcription factor ERF095 OS=Arabidopsis thaliana ON. D=3702 GN=ERF095 PE=1 SV=1 && PF00847:AP2 domain
FaAP2-49	N.D	N.D	N.D	N.D	N.D	5,91	N.D	N.D	|O22259|ERF71_ARATH Ethylene-responsive transcription factor ERF071 OS=Arabidopsis thaliana ON. D=3702 GN=ERF071 PE=1 SV=1 && PF00847:AP2 domain
FaAP2-50	N.D	N.D	N.D	N.D	N.D	60,18	N.D	N.D	|P93835|DRE1B_ARATH Dehydration-responsive element-binding protein 1B OS=Arabidopsis thaliana ON. D=3702 GN=DREB1B PE=1 SV=2 && PF00847:AP2 domain
FaAP2-51	N.D	N.D	N.D	N.D	N.D	51,55	N.D	N.D	|Q9C9I8|ERF20_ARATH Ethylene-responsive transcription factor ERF020 OS=Arabidopsis thaliana ON. D=3702 GN=ERF020 PE=2 SV=1 && PF00847:AP2 domain
FaAP2-52	N.D	N.D	N.D	N.D	N.D	982,65	N.D	N.D	|Q9C9I2|ERF21_ARATH Ethylene-responsive transcription factor ERF021 OS=Arabidopsis thaliana ON. D=3702 GN=ERF021 PE=2 SV=1 && PF00847:AP2 domain
FaAP2-53	N.D	N.D	N.D	N.D	N.D	18,78	N.D	N.D	|Q40479|ERF2_TOBAC Ethylene-responsive transcription factor 2 OS=Nicotiana tabacum ON. D=4097 GN=ERF2 PE=2 SV=1 && PF00847:AP2 domain
FaAP2-54	N.D	N.D	N.D	N.D	N.D	63,20	N.D	N.D	|Q8L9K1|ERF99_ARATH Ethylene-responsive transcription factor 13 OS=Arabidopsis thaliana ON. D=3702 GN=ERF13 PE=1 SV=2 && PF00847:AP2 domain

FaAP2-55	N.D	N.D	N.D	N.D	N.D	333,20	N.D	N.D	ǀO82339ǀCRF5_ARATH Ethylene-responsive transcription factor CRF5 OS=Arabidopsis thaliana ON. D=3702 GN=CRF5 PE=1 SV=1 && PF00847:AP2 domain
FaAP2-56	N.D	N.D	N.D	N.D	N.D	7,28	N.D	N.D	ǀQ9FK12ǀCRF3_ARATH Ethylene-responsive transcription factor CRF3 OS=Arabidopsis thaliana ON. D=3702 GN=CRF3 PE=1 SV=1 && PF00847:AP2 domain
FaAP2-57	N.D	N.D	N.D	N.D	N.D	N.D	2,46	4,06	ǀP47927ǀAP2_ARATH Floral homeotic protein APETALA 2 OS=Arabidopsis thaliana ON. D=3702 GN=AP2 PE=1 SV=1 && PF00847:AP2 domain
FaAP2-58	N.D	N.D	N.D	N.D	N.D	N.D	4,19	N.D	ǀB8AN.DC3ǀAP21_ORYSI APETALA2-like protein 1 OS=Oryza sativa subsp. indica ON.D=39946 GN=AP2-1 PE=2 SV=2 && PF00847:AP2 domain
FaAP2-59	N.D	N.D	N.D	N.D	N.D	N.D	5,91	N.D	ǀQ9SK03ǀRAP27_ARATH Ethylene-responsive transcription factor RAP2-7 OS=Arabidopsis thaliana ON. D=3702 GN=RAP2-7 PE=2 SV=2 && PF00847:AP2 domain
FaAP2-60	N.D	N.D	N.D	N.D	N.D	N.D	31,28	N.D	ǀQ9LYD3ǀDREB3_ARATH Dehydration-responsive element-binding protein 3 OS=Arabidopsis thaliana ON. D=3702 GN=DREB3 PE=2 SV=1 && PF00847:AP2 domain
FaAP2-61	N.D	N.D	N.D	N.D	N.D	N.D	2,97	N.D	ǀO65665ǀERF60_ARATH Ethylene-responsive transcription factor ERF060 OS=Arabidopsis thaliana ON. D=3702 GN=ERF060 PE=1 SV=1 && PF00847:AP2 domain
FaAP2-62	N.D	N.D	N.D	N.D	N.D	N.D	4,42	N.D	ǀQ9C7W2ǀERF61_ARATH Ethylene-responsive transcription factor ERF061 OS=Arabidopsis thaliana ON. D=3702 GN=ERF061 PE=2 SV=1 && PF00847:AP2 domain
FaAP2-64	N.D	N.D	N.D	N.D	N.D	N.D	N.D	3,19	ǀQ6PQQ3ǀAIL5_ARATH AP2-like ethylene-responsive transcription factor AIL5 OS=Arabidopsis thaliana ON. D=3702 GN=AIL5 PE=2 SV=2 && PF00847:AP2 domain
FaAP2-65	N.D	N.D	N.D	N.D	N.D	N.D	N.D	2,01	ǀQ6J9S1ǀRA211_ARATH Ethylene-responsive transcription factor RAP2-11 OS=Arabidopsis thaliana ON. D=3702 GN=RAP2-11 PE=2 SV=1 && PF00847:AP2 domain
FaAP2-66	5,31	N.D	N.D	N.D	8,84	N.D	N.D	2,25	ǀP42736ǀRAP23_ARATH Ethylene-responsive transcription factor RAP2-3 OS=Arabidopsis thaliana ON. D=3702 GN=RAP2-3 PE=1 SV=2 && PF00847:AP2 domain

Table S2. Predicted subcellular localization of FaAP2/ERF transcription factors by *LOCALIZER 1.0.4 Predictions*. Predicted subcellular localization of FaAP2/ERF proteins in chloroplasts, mitochondria, and nucleus. Prediction scores and transit peptide coordinates are shown for organellar localization, and predicted nuclear localization signal (NLS) motifs are provided where applicable. (Y, predicted localization; –, no predicted localization.)

Identifier	Chloroplast	Mitochondria	Nucleus
**FaAP2-1**	-	-	Y(KKFKETRHPVYRGVRRRNS, RRNSGKWVCEVREPNKKTR, KKRAGRKKFKETRHPVYRGVRRRN)
**FaAP2-4**	-	-	-
**FaAP2-7**	-	-	-
**FaAP2-8**	-	-	Y(RRRR, RRRYRGVRQRP)
**FaAP2-9**	-	-	-
**FaAP2-11**	-	-	-
**FaAP2-12**	-	-	Y(KRPWGRYAAEIRDPGKKTR)
**FaAP2-13**	-	-	Y(KRPWGRYAAEIRDPGKKSR)
**FaAP2-14**	-	-	Y(KKRKAGRKKF)
**FaAP2-15**	Y (0.996 ∣ 1-21)	-	Y(KRKR, RKRK, RRIRGKKAKVN)
**FaAP2-16**	-	-	Y(KRKKR, RKWGKWVAEIREPNKRTRI)
**FaAP2-17**	-	-	Y(KRSYGFDSNGKRRARG, KRMREFELLELEFSKKQRI)
**FaAP2-18**	-	-	Y(RKRRR)
**FaAP2-19**	-	-	Y(RKRRR, SSRKR)
**FaAP2-20**	-	-	Y(KPRK, RRHSMRRK)
**FaAP2-21**	-	-	Y(RKRRR, RRREEEVQPEVPVVVKKEK, RREEEVQPEVPVVVKKEKV)
**FaAP2-22**	-	-	Y(KRKHSMRRKMGNKKSKM)
**FaAP2-23**	-	-	Y(PDSDPARKKHFRGVRQRPWG)
**FaAP2-24**	-	-	Y(PKRRK, KRRKK, KRSSPEPASSSESGSPKRR)
**FaAP2-25**	-	-	Y(KKGPKKRVKKNYRGVRQRPWGKWAAEIR)
**FaAP2-26**	-	-	-
**FaAP2-27**	-	-	Y(RKRRK)
**FaAP2-28**	-	-	-
**FaAP2-29**	-	-	Y(KSKRAR)
**FaAP2-30**	-	-	-
**FaAP2-31**	-	-	Y(KRRWGKWVSEIREPRKKSR, RRWGKWVSEIREPRKKSRI, GVRKRRWGKWVSEIREPRKKS)
**FaAP2-32**	-	Y(0.947∣1-22)	-
**FaAP2-33**	-	-	Y(RKKTAGKTVAPVTHRTLKPAPGKKFRGVRQRPWGK)
**FaAP2-34**	-	-	-
**FaAP2-35**	-	-	Y(VGKKHYRGVRRRPW)
**FaAP2-36**	-	-	-
**FaAP2-37**	-	-	Y(KPKPKRSRKTPNK)
**FaAP2-38**	-	-	Y(KRIR,KRNCIDVDDSEKTRRC,RRCGTEYKGVRRRKWGK, KTKSNLKKLHLSKKAKG, KKRVKFDNRRLLKKTKSNLKKLHLSKKAK)
**FaAP2-39**	-	-	Y(RKSK, RGIRRR)
**FaAP2-42**	-	-	Y(RASKRRS, LKFKLKLKKG)
**FaAP2-43**	-	-	Y(RQRP)

**FaAP2-44**	-	-	Y(KKRHSMKRKSAIKKKKKNV)
**FaAP2-45**	Y(0.876∣1-30)	-	Y(KKPR, KRPWGRFAAEIRDPWKKTR)
**FaAP2-46**	-	-	Y(KKGPKKRVKKNYRGVRQRPWGKWAAEIR)
**FaAP2-47**	-	-	-
**FaAP2-48**	-	-	-
**FaAP2-49**	-	-	Y(RKIRGKKA)
**FaAP2-50**	-	-	Y(RRSAHSDEEVILASSRPKK, RRVFKETRHPVYRGVRRRN, RRNNNKWVCEVREPNKKTR, RPKKRAGRRVFKETRHPVYRGVRRR)
**FaAP2-51**	-	-	-
**FaAP2-52**	-	-	Y(RKRKWGK, KRKWGKWVSEIREPGKKTR, RKWGKWVSEIREPGKKTRI)
**FaAP2-53**	-	-	Y(PKPKRMR, RRRPWGKYAAEIRDPKK)
**FaAP2-54**	-	-	Y(PKPKRRRR, RRRPWGKYSAEIRDPKKKG, RRPWGKYSAEIRDPKKKGM)
**FaAP2-55**	-	-	Y(KKKFRGVRQRPWGKWA)
**FaAP2-56**	-	-	-
**FaAP2-57**	-	-	-
**FaAP2-58**	-	-	Y(PPPPQVKKSRRGPRSR)
**FaAP2-59**	-	-	Y(SRRG)
**FaAP2-60**	-	-	Y(KRAR)
**FaAP2-61**	-	-	-
**FaAP2-62**	-	-	Y(KVKK, VKKAKKKNKS)
**FaAP2-64**	-	-	-
**FaAP2-65**	-	-	Y(PKKAK)
**FaAP2-66**	-	-	Y(KRIR,KRSR,KKKTTIGIAEKEADGKTKR, KKTTIGIAEKEADGKTKRV, GIAEKEADGKTKRVRKNKYRGIRQRPWGK)

Proteins analyzed: 58

Number of proteins with cTP: 0 (0.0%)
Number of proteins with cTP & possible mTP: 0 (0.0%)
Number of proteins with cTP & NLS: 2 (3.4%)
Number of proteins with cTP & possible mTP & NLS: 0 (0.0%)
Number of proteins with mTP: 1 (1.7%)
Number of proteins with mTP & possible cTP: 0 (0.0%)
Number of proteins with mTP & NLS: 0 (0.0%)
Number of proteins with mTP & possible cTP & NLS: 0 (0.0%)
Number of proteins with NLS and no transit peptides: 41 (70,7%)

Summary statistics

Number of proteins with chloroplast localization (cTP, cTP & possible mTP, cTP & NLS, cTP & possible mTP & NLS): 2 (3.4%)
Number of proteins with mitochondrial localization (mTP, mTP & possible cTP, mTP & NLS, mTP & possible cTP & NLS): 1 (1.7%)
Number of proteins with nuclear localization and no transit peptides: 41 (70,7%)
Number of proteins with nuclear localization and with transit peptides: 2 (3.4%)

Table S3. Expression pattern-based clustering of *FaAP2/ERF* genes in response to drought and salinity stress. *FaAP2/ERF* genes were classified into upregulated, downregulated, and mixed expression clusters according to their qualitative RT-qPCR expression profiles in leaves and roots under drought and salinity conditions. Genes displaying identical expression patterns across the four treatments were assigned to the same cluster. The table lists the expression profile, corresponding gene IDs, and the number of genes included in each cluster

Expression Cluster UP REGULATED	Expression profile	Gene IDs	Number of genes
Cluster 1	Upregulated in drought leaf, drought root, salinity leaf and salinity root	*FaAP2-1, FaAP2-4, FaAP2-7, FaAP2-8, FaAP2-9, FaAP2-13, FaAP2-14, FaAP2-20, FaAP2-22, FaAP2-24, FaAP2-25, FaAP2-26, FaAP2-35, FaAP2-39, FaAP2-43, FaAP2-44, FaAP2-47, FaAP2-52, FaAP2-53, FaAP2-54*	20
Cluster 2	Upregulated in drought root, salinity leaf and salinity root	*FaAP2-51, FaAP2-28, FaAP2-49*	3
Cluster 3	Upregulated in drought leaf, drought root and salinity root	*FaAP2-16, FaAP2-21*	2
Cluster 4	Upregulated in drought leaf, salinity leaf and salinity root	*FaAP2-19, FaAP2-23, FaAP2-46, FaAP2-50*	4
Cluster 5	Upregulated in drought leaf and salinity root	*FaAP2-12, FaAP2-45, FaAP2-62*	3
Cluster 6	Upregulated in drought leaf and salinity leaf	*FaAP2-65*	1
Cluster 7	Upregulated in drought leaf and root	*FaAP2-18*	1
Cluster 8	Upregulated in salinity leaf and salinity root	*FaAP2-36, FaAP2-48*	2
Cluster 9	Upregulated exclusively in drought leaf	*FaAP2-66, FaAP2-15*	2
Cluster 10	Upregulated exclusively in salinity root	*FaAP2-42*	1
**Expression Cluster DOWN REGULATED**	**Expression profile**	**Gene IDs**	**Number of genes**
Cluster 1	Downregulated in both drought leaf and salinity leaf	*FaAP2-30*	1
Cluster 2	Downregulated exclusively in salinity leaf and roots	*FaAP2-29*	1

Cluster 3	Downregulated exclusively in salinity leaf	*FaAP2-33, FaAP2-38, FaAP2-58*	3
**Expression Cluster MIXED**	**Expression profile**	**Gene IDs**	**Number of genes**
Mixed 1	Upregulated in drought leaf, salinity leaf and salinity root; downregulated in drought root	*FaAP2-11*	1
Mixed 2	Upregulated in drought leaf and roots and salinity root, downregulated in salinity leaf	*FaAP2-17,*	1
Mixed 3	Upregulated in drought leaf and salinity root; downregulated in drought root	*FaAP2-61*	1
Mixed 4	Upregulated in drought and salinity root; downregulated in salinity leaf	*FaAP2-27, FaAP2-31,*	2
Mixed 5	Upregulated in salinity leaf and root; downregulated in drought leaf	*FaAP2-55, FaAP2-37*	2
Mixed 6	Upregulated in salinity root; downregulated in drought leaf and root and salinity root	*FaAP2-34*	1
Mixed 7	Upregulated in drought root; downregulated in drought leaf and salinity leaf	*FaAP2-64*	1
Mixed 8	Upregulated in salinity root; downregulated in drought root and salinity leaf	*FaAP2-56*	1
Mixed 9	Upregulated in drought root; downregulated in salinity leaf	*FaAP2-59*	1
Mixed 10	Upregulated in salinity leaf and downregulated in salinity root	*FaAP2-32*	1
Mixed 11	Upregulated in salinity root and downregulated in salinity leaf	*FaAP2-57, FaAP2-60*	2

Table S5. Oligonucleotide primers used for quantitative real-time PCR (RTq-PCR). The table lists the forward (Fw) and reverse (Rv) primer sequences (5′–3′) designed for the amplification of *Fragaria × ananassa AP2/ERF* genes, together with the reference genes *FaGAPDH2* and *FaEF1α* used for gene expression normalization.

ID	OLIGOS 5'-->3'
**FaAP2_1-qRT-Fw**	GCCAGTGCCGGCTTCTAATA
**FaAP2_1-qRT-Rv**	TCCCAAACACATCCTCCTCG
**FaAP2-2-qRT-Fw**	GCCTAATGGGGAGGAATCGG
**FaAP2-2-qRT-Rv**	AACCTTCCATGTCATCGCCG
**FaAP2-3-qRT-Fw**	TCTCCCTAAGCAGTTGCAGG
**FaAP2-3-qRT-Rv**	GCCTCCGTGAGGGTATGAGA
**FaAP2-4-qRT-Fw**	GGCACAAAAGCCAAGCTCAA
**FaAP2-4-qRT-Rv**	GGGACGGTGCAACATAGGAA
**FaAP2-5-qRT-Fw**	CTCCAGAAGTTCAGCGACCA
**FaAP2-5-qRT-Rv**	CATTTGCCCCAATGCCTCTG
**FaAP2-6-qRT-Fw**	TGGATAGGTCAGGTGGTGGT
**FaAP2-6-qRT-Rv**	GCGAATCATTAGCGGGTCCT
**FaAP2-7-qRT-Fw**	TCGTCTCAAAACCACGGACA
**FaAP2-7-qRT-Rv**	CCGCCAAATGGCATAATGGG
**FaAP2-8-qRT-Fw**	GTCCCGGTGGACTATGAGGA
**FaAP2-8-qRT-Rv**	CCGATACGACATGCGTCAGT
**FaAP2-9-qRT-Fw**	GATTTGAATTCGTCGCCGGG
**FaAP2-9-qRT-Rv**	CGAGGAAGATGAACCCTGCT
**FaAP2_10-qRT-Fw**	TGGATAGGTCAGGTGGTGGT
**FaAP2_10-qRT-Rv**	GCGAATCATTAGCGGGTCCT
**FaAP2_11-qRT-Fw**	GGGAGAAATGGCAGAGGCTT
**FaAP2_11-qRT-Rv**	CCTGTTGCGGAAAGTCATGC
**FaAP2_12-qRT-Fw**	GACCAGAACGTGGTGACGAT
**FaAP2_12-qRT-Rv**	GACGAGTCGGAGTCGCTAAC
**FaAP2_13-qRT-Fw**	TAACAACGGCGGGAATAGCA
**FaAP2_13-qRT-Rv**	GGAATCTGGTGATGGGGGTG
**FaAP2_14-qRT-Fw**	TCAAGAAGAGGAAAGCCGGG
**FaAP2_14-qRT-Rv**	ACAAGTAAAGGTCCCGAGCC
**FaAP2_15-qRT-Fw**	AGGACTCCTGAAATCTCATCTGT
**FaAP2_15-qRT-Rv**	ATCCCAGCTCCCCTCAAGAT
**FaAP2_16-qRT-Fw**	TCCATACGCAAGAAGGCCAC
**FaAP2_16-qRT-Rv**	GGTCCGGGTACTCGTTCAAA
**FaAP2_17-qRT-Fw**	CCGGACAGGCAGCTTTAT
**FaAP2_17-qRT-Rv**	GCCTCCACAGTAGTACCAGC
**FaAP2_18-qrt-Fw**	CGTAGTTGGCCGGAAGAGG
**FaAP2_18-qrt-Rv**	GAGGTGCTGTTACCACTGCT
**FaAP2_21-qrt-Fw**	CCTCTAGAAGCCGGGAAATATGA
**FaAP2_21-qrt-Rv**	AACGGCGGAACACTGAAGAT
**FaAP2_22-qrt-Fw**	CAACGAAAACGACTCCGAGG
**FaAP2_22-qrt-Rv**	AGGCAGCTGAGTTGTGTGTT
**FaAP2_23-qrt-Fw**	GAGACCGTTGTTGCTGAGGA

**FaAP2_23-qrt-Rv**	GCACGTCGTATTCAAACCCG
**FaAP2_24-qrt-Fw**	ATGGGGAGTGGGATTGGGAT
**FaAP2_24-qrt-Rv**	CAAGTACTGCTTCCCAGCTCA
**FaAP2_25-qrt-Fw**	AACGGTGGATGATGACGGAC
**FaAP2_25-qrt-Rv**	TGATGCAAACCTCTGGTGCT
**FaAP2_26-qrt-Fw**	TGGTTCTCATACCCTCACGG
**FaAP2_26-qrt-Rv**	CACTGCAGCTCCTGTCTTACT
**FaAP2_27-qrt-Fw**	AGAGTCTAACCCGCCGAGTA
**FaAP2_27-qrt-Rv**	GCAGAGCATCCTGAGAACCC
**FaAP2_28-qrt-Fw**	GAAGCGCTGCTGAATATGCC
**FaAP2_28-qrt-Rv**	CTGTGACACCTTCCATGCCA
**FaAP2_29-qrt-Fw**	CACCAGTTCGACTCGACCAA
**FaAP2_29-qrt-Rv**	TCGTAACTCGTCCCCAGACT
**FaAP2_30-qrt-Fw**	CTCGGGCTATCAAAGGTGCT
**FaAP2_30-qrt-Rv**	TTGCTGGGTTGTGTTGCTTG
**FaAP2_31-qrt-Fw**	ATGGCCAGAAGGAGAGGCAT
**FaAP2_31-qrt-Rv**	GGAACAACCCCGAGAGACAC
**FaAP2_32-qrt-Fw**	AGTCAAAGTGGAGAGCACCC
**FaAP2_32-qrt-Rv**	TGGCTGAGACCGATTAAGGT
**FaAP2_33-qrt-Fw**	ATTTCGGGTTCGGATCCTCC
**FaAP2_33-qrt-Rv**	AAGAAACAGCGGTGGGAGAG
**FaAP2_34-qrt-Fw**	CTGCCTGGACTGATGCTTGA
**FaAP2_34-qrt-Rv**	TCCAATGCTTTCACTCCCTCA
**FaAP2_35-qrt-Fw**	CACGTTTAACACGGCCGAAG
**FaAP2_35-qrt-Rv**	AAGCTTGGCGCAACTCTTTG
**FaAP2_36-qrt-Fw**	CTATGCAGAAGGGGACGACG
**FaAP2_36-qrt-Rv**	GCCCTCCGTCCATATTCACA
**FaAP2_37-qrt-Fw**	TGTGCCGGAAAACTTGGATG
**FaAP2_37-qrt-Rv**	ACATGTTTTCGATTCCCTGCC
**FaAP2_38-qrt-Fw**	CGTGCGATGATGTGAATGGG
**FaAP2_38-qrt-Rv**	CCGTCATACCTGCAACCACA
**FaAP2_39-qrt-Fw**	TTGGAAACGAGGAAGGTCGAT
**FaAP2_39-qrt-Rv**	CCCACCGCCATCATTGTAATTG
**FaAP2_40-qrt-Fw**	TTTCCCACCGTTGGATGAGG
**FaAP2_40-qrt-Rv**	ACCAAGGATGTTTGAAGTGTGT
**FaAP2_41-qrt-Fw**	GGATGATAGGCTGCTGGAGG
**FaAP2_41-qrt-Rv**	CGGATGAGCACAAATTCGAAGA
**FaAP2_42-qrt-Fw**	AACTTGACTGGGAGCACTGT
**FaAP2_42-qrt-Rv**	TACGTGCCCTGCATCACTG
**FaAP2_43-qrt-Fw**	CTCCTATGCCGGATTTCGGT
**FaAP2_43-qrt-Rv**	TCGATTCCAAGGTCGATCACT
**FaAP2_44-qrt-Fw**	GTGCGAGTTTGGTTGGGAAC
**FaAP2_44-qrt-Rv**	CAAAACCGGAGATGAGCCCT
**FaAP2_45-qrt-Fw**	CGGTGTATCCTGTTTCGCCT
**FaAP2_45-qrt-Rv**	AGATCGAAGGGCAGGACTCT
**FaAP2_46-qrt-Fw**	GTCGGATTCTGGAAACGGGT

**FaAP2_46-qrt-Rv**	ACACTATGTGATTTCCCCTCAA
**FaAP2_47-qrt-Fw**	CTGCTGCTGATGACACTGGG
**FaAP2_47-qrt-Rv**	CATGCAAATCAATCAACCTGAAACT
**FaAP2_48-qrt-Fw**	GCCATGGGGAAAGTTTGCTG
**FaAP2_48-qrt-Rv**	AAGTAGCAGCAGCGGAATCA
**FaAP2_49-qrt-Fw**	GCTTTCTGAGGAGCTAATGGC
**FaAP2_49-qrt-Rv**	AAGAGGTGACAGAAGCAGGAA
**FaAP2_50-qrt-Fw**	ATCGACAAGCCGGAATCCTC
**FaAP2_50-qrt-Rv**	GTGGCGAGTCTCCTTGAACA
**FaAP2_51-qrt-Fw**	GTTCAACCACTAACAGCGGC
**FaAP2_51-qrt-Rv**	GCCGGAATAGACAGCCTACC
**FaAP2_52-qrt-Fw**	TGACTTCGACGATGAGGGGA
**FaAP2_52-qrt-Rv**	AGGTCAAACTTGGTCACACCT
**FaAP2_53-qrt-Fw**	GCCGGAGAGGTTCATGTGTT
**FaAP2_53-qrt-Rv**	ATCTACAATTTTCTTCTGCCGGA
**FaAP2_54-qrt-Fw**	TCGATCACATTTGGCGACGA
**FaAP2_54-qrt-Rv**	GGAAAATGGAACCAGAACAAGC
**FaAP2_55-qrt-Fw**	TTGTCCGACATTTCGCTGGA
**FaAP2_55-qrt-Rv**	ACACAGAGATTTACAGAGGGGA
**FaAP2_56-qrt-Fw**	GTTCCCGGACCACTCATGTT
**FaAP2_56-qrt-Rv**	ACTGCCTAGTGTCAACTGCC
**FaAP2_57-qrt-Fw**	CGACAACAATGGCAACCTGG
**FaAP2_57-qrt-Rv**	GGGTGATCTCCCTAGTCCAA
**FaAP2_58-qrt-Fw**	CACTGTTCTCTACCGCAGCA
**FaAP2_58-qrt-Rv**	TATGGACGCAATCGGCTACC
**FaAP2_59-qrt-Fw**	TACCGTACCTCCATCAGCCA
**FaAP2_59-qrt-Rv**	AGTACTGCTGCACATTACAGACT
**FaAP2_60-qrt-Fw**	TTGAGGACCTGTTGTGGCAA
**FaAP2_60-qrt-Rv**	TGAGAATCACACACTCAAAACACA
**FaAP2_61-qrt-Fw**	CGCAGCTCTAGCTCCACTAC
**FaAP2_61-qrt-Rv**	CCCCTGTAGAGCTTTGTGGG
**FaAP2_62-qrt-Fw**	ACGCCAAGATTCAGGCCATT
**FaAP2_62-qrt-Rv**	CGAGGTCGAGGAAGATGTCG
**FaAP2_63-qrt-Fw**	GGAAAGGACGTCAAGGTGGAT
**FaAP2_63-qrt-Rv**	CTTGCTTGGTCACCAGCTTC
**FaAP2_64-qrt-Fw**	ACTCCCATTTTCGGCATGGA
**FaAP2_64-qrt-Rv**	TGCCCAGTAGGTGTCAGTCT
**FaAP2_65-qrt-Fw**	CAGCCTAGAGAGCTCAACACC
**FaAP2_65-qrt-Rv**	GATCTCAGCCACCCACCTTC
**FaAP2-66qrt-Fw**	AAGGCCAAGCTCAACTTCCC
**FaAP2-66-qrt-Rv**	GGTCGTGATCAGCCGTAGTC
**FaGAPDH2-qrt-Fw**	CCCAAGTAAGGATGCCCCCATGTTCG
**FaGAPDH2-qrt-Rv**	TTGGCAAGGGGAGCAAGACAGTTGGTAG
**FaEF1α-qrt-Fw**	TGGATTTGAGGGTGACAACATGA
**FaEF1α -qrt-Rv**	GTATACATCCTGAAGTGGTAGACGGAG
**NbU74E2-qrt-Fw**	TTCGTTGCCTTCTCTTCCGT

**NbU74E2-qrt-Rv**	CCTCCTCCTCCAACATGTCAAA
**NbNAC6-qrt-Fw**	GGGAGGCCTAACAGAACAACA
**NbNAC6-qrt-Rv**	AGGTGCCCTTCCACCATAGA
**NbHIR1-qrt-Fw**	GCACGACAACGTCAAGCAAT
**NbHIR1-qrt-Rv**	TTGCTGGAAGCGCCAATTTC
**NbSUS2-qrt-Fw**	CGTCCAGCCTGCATTTTACG
**NbSUS2-qrt-Rv**	AAGTTCAGCTGCCTGATCCC
**NbU91A1-qrt-Fw**	TCACTGCTTCTTCACGGGTC
**NbU91A1-qrt-Rv**	AGCAACCATCGACGGGAAAT
**NbOSCA1.2-qrt-Fw**	CAGTGGTCACACCCGTACTC
**NbOSCA1.2-qrt-Rv**	TGCTCAACAGTCCCAGCAAT
**NbCML38-qrt-Fw**	GATGGCGGTGAGACTATCGG
**NbCML38-qrt-Rv**	AGTAACGTAGCCACTCCCCT
**NbDREB1A-qrt-Fw**	TAACCCCAAGAAGCGAGCAG
**NbDREB1A-qrt-Rv**	GAGCTCTAGCCGCCATTTCT
**NbPUB19-qrt-Fw**	ATCTTCACCCCGCGAATACC
**NbPUB19-qrt-Rv**	TATGACTCAGCAGCAACCCG
**NbCML37-qrt-Fw**	GTGTGTCAAGGCGGTAGGAG
**NbCML37-qrt-Rv**	TGTAGCCACTCCCCTCCATT
**NbUBQ3-qrt-Fw**	ACGGCATAGAGATGGGTCAC
**NbUBQ3-qrt-Rv**	CCTTCTTCAATCCCTCACCA
